# Functionally distinct POMC-expressing neuron subpopulations in hypothalamus revealed by intersectional targeting

**DOI:** 10.1038/s41593-021-00854-0

**Published:** 2021-05-17

**Authors:** Nasim Biglari, Isabella Gaziano, Jonas Schumacher, Jan Radermacher, Lars Paeger, Paul Klemm, Weiyi Chen, Svenja Corneliussen, Claudia M. Wunderlich, Michael Sue, Stefan Vollmar, Tim Klöckener, Tamara Sotelo-Hitschfeld, Amin Abbasloo, Frank Edenhofer, Frank Reimann, Fiona M. Gribble, Henning Fenselau, Peter Kloppenburg, Frank T. Wunderlich, Jens C. Brüning

**Affiliations:** 1 Max Planck Institute for Metabolism Research, Department of Neuronal Control of Metabolism, Cologne, Germany; 2 Policlinic for Endocrinology, Diabetes and Preventive Medicine (PEDP), University Hospital Cologne, Cologne, Germany; 3 Excellence Cluster on Cellular Stress Responses in Aging Associated Diseases (CECAD) and Center of Molecular Medicine Cologne (CMMC), University of Cologne, Cologne, Germany; 4 Institute for Zoology, Biocenter, University of Cologne, Cologne, Germany; 5 Leopold-Franzens-Universität Innsbruck, Institute for Molecular Biology, Innsbruck, Austria; 6 Cambridge Institute for Medical Research and Medical Research Council Metabolic Diseases Unit, Addenbrooke’s Hospital, Cambridge, UK; 7 Max Planck Institute for Metabolism Research, Research Group Synaptic Transmission in Energy Homeostasis, Cologne, Germany; 8 National Center for Diabetes Research (DZD), Ingolstädter Landstrasse 1, Neuherberg, Germany

**Keywords:** Neuroscience, Feeding behaviour, Hypothalamus

## Abstract

Pro-opiomelanocortin (POMC)-expressing neurons in the arcuate nucleus of the hypothalamus represent key regulators of metabolic homeostasis. Electrophysiological and single-cell sequencing experiments have revealed a remarkable degree of heterogeneity of these neurons. However, the exact molecular basis and functional consequences of this heterogeneity have not yet been addressed. Here, we have developed new mouse models in which intersectional Cre/Dre-dependent recombination allowed for successful labeling, translational profiling and functional characterization of distinct POMC neurons expressing the leptin receptor (*Lepr*) and glucagon like peptide 1 receptor (*Glp1r*). Our experiments reveal that POMC^Lepr+^ and POMC^Glp1r+^ neurons represent largely nonoverlapping subpopulations with distinct basic electrophysiological properties. They exhibit a specific anatomical distribution within the arcuate nucleus and differentially express receptors for energy-state communicating hormones and neurotransmitters. Finally, we identify a differential ability of these subpopulations to suppress feeding. Collectively, we reveal a notably distinct functional microarchitecture of critical metabolism-regulatory neurons.

## Abstract (web summary)

Biglari et al. reveal subgroups of arcuate nucleus hypothalamic neurons that exhibit distinct molecular signatures and feeding-regulatory functions, thus uncovering new regulatory principles in body weight control.

## Main

The melanocortin circuitry comprising agouti-related peptide (AgRP)-expressing neurons and POMC-expressing neurons in the arcuate nucleus of the hypothalamus (ARC) represents a prototypic homeostatic regulatory neurocircuit of metabolic homeostasis^1^. These targets of insulin, leptin and glucagon like peptide (Glp1) integrate multiple inputs to compute the energy state of the organism and adapt feeding behavior^2^. The POMC pro-peptide is processed to α-melanocyte-stimulating hormone, which is an activator of the melanocortin 4 receptor^3^ to suppress food intake^4^. Conversely, AgRP released from AgRP neurons acts as an inverse agonist on the melanocortin 4 receptor, thereby promoting feeding^5^. In addition, the melanocortin circuitry is an integrative regulator of numerous physiological functions. For instance, insulin action in AgRP neurons is required to efficiently suppress hepatic glucose production, and abrogation of insulin and leptin receptors from POMC neurons causes diabetes in mice^6^. Further, acute chemogenetic or optogenetic activation of AgRP neurons controls insulin sensitivity^7^. Thus, the melanocortin circuitry integrates the energy state of an organism to correspondingly adapt food intake as well as substrate flux across different organs^8^.

Although POMC neurons have been considered a homogeneous cell group, previous studies have highlighted their functional diversification. Specifically, 30% of POMC neurons increase firing in response to leptin in brain slice electrophysiology, and this response does not overlap with POMC cells in which insulin modulates firing^9^. Similarly, some POMC neurons express GABAergic markers and others express glutamatergic markers^10^. Moreover, studies using single-cell mRNA sequencing (RNA-seq) revealed a striking molecular heterogeneity of these neurons^11,12^. However, the molecular basis and associated functional consequences of their heterogeneity remain unknown.

Modern molecular systems neuroscience has been revolutionized through the ability to characterize neuronal subtypes on a molecular level at single-cell resolution and to functionally investigate distinct neurocircuits through employment of molecular tools for cell-type-specific manipulations. Here, Cre-loxP-mediated recombination provides a critical repertoire for cell-type-specific inactivation or activation of genes or transgenes^13^. These transgenes may represent genetically encoded fluorophores, expression of chemogenetically modifiable designer G-protein-coupled receptors, hM3Dq or light-regulated ion channels for remotely controlled activation or inhibition of Cre-expressing neurons in vivo^14,15^. To address the question of cellular heterogeneity, combinatorial recombinase-dependent targeting of distinct cell types using two different, intersectional recombinases such as Cre and FLP have been successfully developed^16^. Through the availability of an additional, complementary recombinase system that builds on expression of Dre-recombinase, an alternative and complementary approach has been defined^17^.

We have dedicated our efforts toward further developing a toolbox of transgenic mice that offers the opportunity to genetically dissect heterogeneous neuron populations, through combinatorial Cre-dependent and Dre-dependent recombination, allowing for successful labeling, three-dimensional (3D) imaging, translational profiling and functional characterization of POMC neurons expressing Lepr or Glp1r, uncovering a new organizational and functional microarchitecture of critical metabolism-regulatory neurons.

## Results

### Generation of POMC^Dre^-transgenic mice

**Fig. 1:**
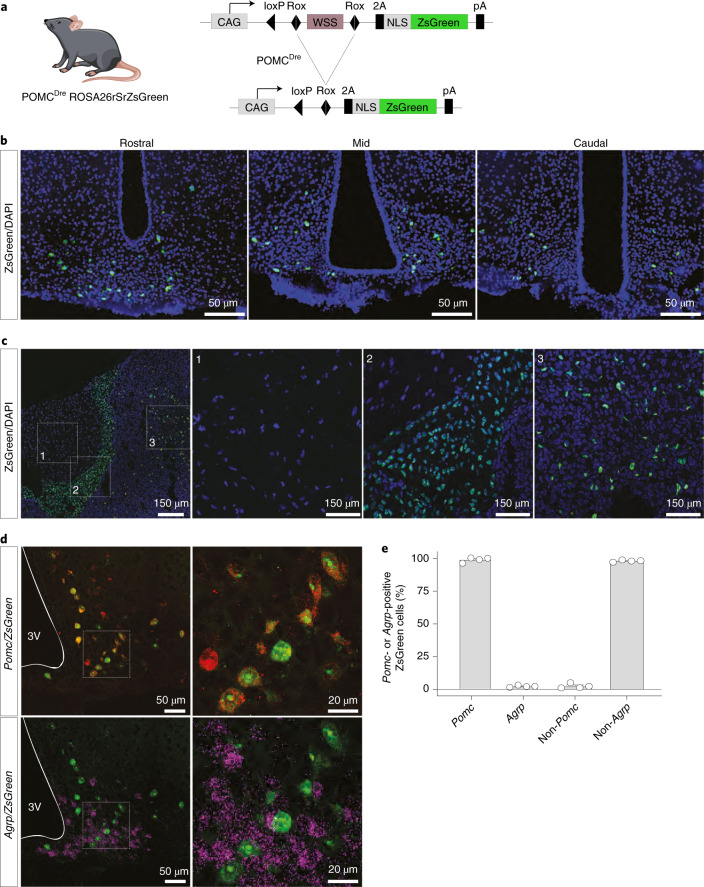
The POMC^Dre^ driver line successfully targets POMC neurons. **a**, Schematic showing POMC^Dre^-dependent recombination in the ROSA26rSrZsGreen reporter line. Excision of *rox*-flanked stop cassette leads to ZsGreen expression in POMC neurons. **b**, ZsGreen expression across the rostral, mid and caudal sections of the ARC in POMC^Dre^ ROSA26rSrZsGreen mice at 15 weeks of age. **c**, Dispersed ZsGreen expression in the intermediate and anterior lobes of the pituitary in POMC^Dre^ ROSA26rSrZsGreen mice. C1, C2 and C3 depict magnifications of the posterior, intermediate and anterior pituitary, respectively. Scale bar, 150 μm (whole image) and 50 μm (magnified images). **d**, RNA ISH against *Pomc*/*ZsGreen* (top) and *Agrp*/*ZsGreen* (bottom) in POMC^Dre^ ROSA26rSrZsGreen mice. Magnifications of the boxes are displayed on the right of each image. Scale bars, 50 μm (whole image) and 20 μm (magnified images). 3V, third ventricle. **e**, Percentage of ZsGreen-positive cells coexpressing or lacking expression of either *Pomc* or *Agrp*, quantified from RNA ISH (**d**). Data are represented as the mean ± s.e.m. (*Pomc*: 97.57 ± 0.92; *Agrp*: 1.88 ± 0.36; non-*Pomc*: 2.43 ± 0.92; non-*Agrp*: 98.12 ± 0.36; *n* = 4 mice; a minimum of 13 sections were analyzed for each group).

**Extended Data Fig. 1:**
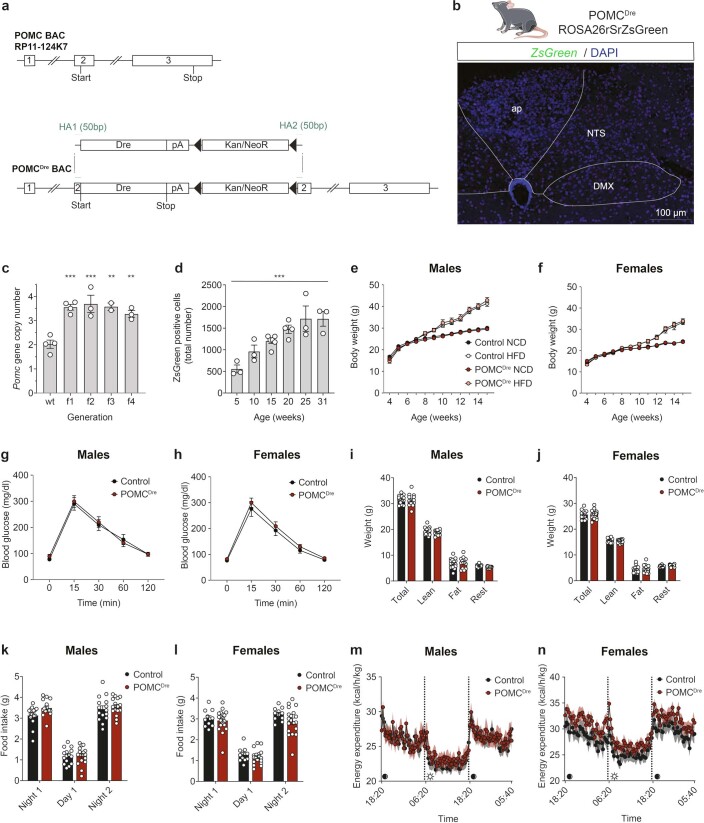
The POMC^Dre^ transgene shows stable integration into the genome and causes no metabolic phenotype as compared to wildtype controls. **a**, Schematic diagram of the targeting strategy for the POMCDre BAC construct. **b**, RNA *in situ* hybridization against *ZsGreen* in POMCDre ROSA26rSrZsGreen mice. Scale bars represent 100 μm. ap = area postrema, NTS = nucleus tractus solitarius, DMX = dorsal motor nucleus. **c**, Quantification of copy number PCR of *Pomc* exon 1, performed on genomic DNA from the first four generations (f1-f4) of the POMC^Dre^ mouse line in comparison to wildtype (wt) animals. For wt, f1, f2, f3, f4; n = 4, 4, 3, 2, 3, respectively. For wt vs. f1 *p* = 0.0004, wt vs. f2 *p* = 0.0003, wt vs. f3 *p* = 0.0016, wt vs. f4 *p* = 0.0031. **d**, Quantification of ZsGreen-positive POMC neurons in the ARC of POMC^Dre^ ROSA26rSrZsGreen mice at indicated time points. For age groups 5, 10, 15, 20, 25 and 31 weeks; n = 3, 3, 6, 4, 3, 3, respectively. *p* = 0.0002. **e-f**, Body weight curve of POMC^Dre^ males (**e**, Control NCD n = 19, Control HFD n = 23, POMC^Dre^ NCD n = 15, POMC^Dre^ HFD n = 4) and females (**f**, Control NCD n = 12, Control HFD n = 8, POMC^Dre^ NCD n = 19, POMC^Dre^ HFD n = 7) versus control littermates on NCD and HFD. **g-n**, Glucose tolerance test (**g**, **h**), body composition (**i**, **j**), food intake (**k**, **l**), and energy expenditure (**m**, **n**) of POMC^Dre^ males and females on NCD. **g**, Control n = 20, POMC^Dre^ n = 16, **h**, Control n = 12, POMC^Dre^ n = 19, **i**, Control n = 17, POMC^Dre^ n = 14, **j**, Control n = 12, POMC^Dre^ n = 19, **k**, Control n = 15, POMC^Dre^ n = 16, **l**, Control n = 11, POMC^Dre^ n = 19, **m**, Control n = 17, POMC^Dre^ n = 14, **n**, Control n = 12, POMC^Dre^ n = 19. n indicates the number of mice. Data are represented as mean ± SEM. *p*-values were calculated by one-way-ANOVA (**d**) followed by Dunnett’s (**c**), two-way-ANOVA (**g**, **h**, **m**, **n**) or unpaired two-tailed Student’s t-test followed by Holm-Sidak correction for multiple comparisons (**i**, **j**, **k**, **l**). *p ≤ 0.05, **p ≤ 0.01, ***p ≤ 0.001. Further statistical details are given in Source data extended Fig. 1. Source data extended Fig. 1. Source data

We generated mice, which express the Dre-recombinase under control of the POMC promoter (POMC^Dre^ mice; Extended Data Fig. 1a). Assessment of Dre-dependent recombination via visualization of Dre-dependent reporter expression revealed ZsGreen-positive cells in the ARC and in the anterior and intermediate lobes of the pituitary for five of seven transgenic lines (Fig. 1a–c). In particular, one line effectively labeled POMC cells in the pituitary and in the ARC (Fig. 1b,c). Double RNA in situ hybridization (ISH) revealed that 97.6% of ZsGreen-positive neurons in the ARC expressed *Pomc*, while only 1.9% expressed *Agrp* (Fig. 1d,e). Correspondingly, the proportion of ZsGreen-labeled non-*Pomc*-expressing cells was 2.4% and that of ZsGreen-labeled non-*Agrp*-expressing cells was 98.1% (Fig. 1d,e). We observed no POMC^Dre^-dependent labeling of ZsGreen-positive neurons in the nucleus tractus solitarius (NTS; Extended Data Fig. 1b). Assessment of the bacterial artificial chromosome (BAC) transgene copy number revealed stable transgenerational copy numbers, indicating a single genomic integration (Extended Data Fig. 1c). Longitudinal assessment of Dre-dependent recombination revealed a continued increase of Dre-dependent recombination in POMC neurons from 3–15 weeks of age (Extended Data Fig. 1d). Thus, the later onset of Dre-dependent recombination in our model allows selective marking of bona fide POMC neurons without substantial recombination in the functionally antagonistic AgRP neurons as present in POMC^Cre^ transgenic mice^18^.

Next, we investigated whether insertion of the BAC transgene in this POMC^Dre^ line resulted in any metabolic phenotype. However, POMC^Dre^ mice did not exhibit any differences in body weight, food intake, locomotor activity, energy expenditure or glucose tolerance compared to their littermate controls (Extended Data Fig. 1e–n).

### Combinatorial recombinase-dependent marking of heterogeneous POMC neuron populations

**Fig. 2:**
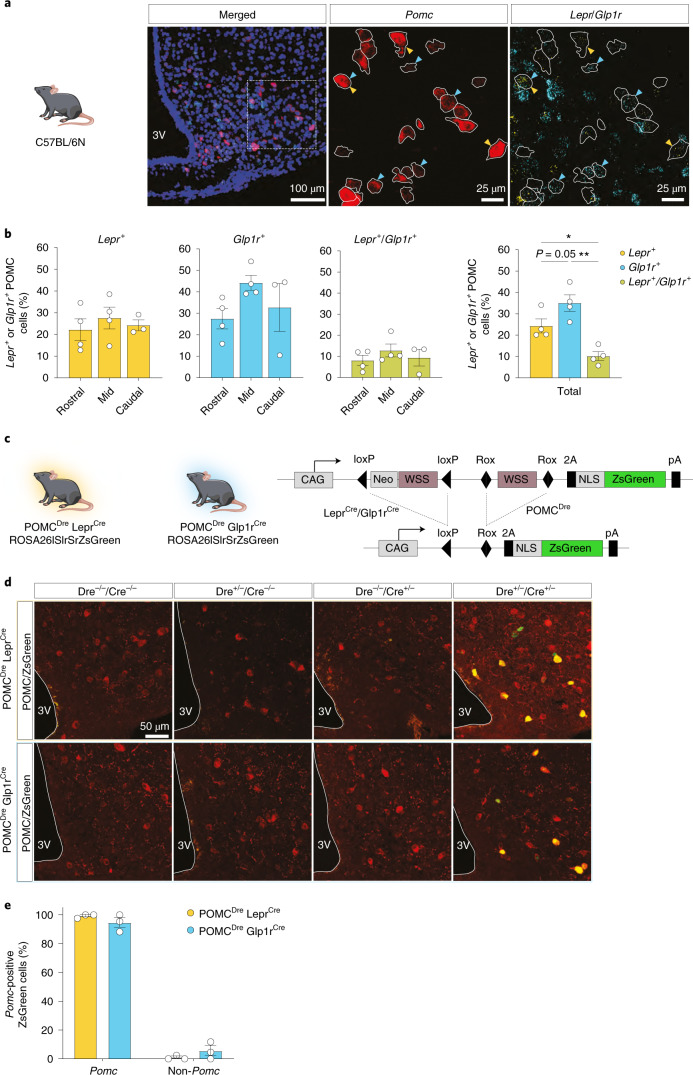
*Lepr* and *Glp1r* expression in POMC neurons. **a**, Representative microscopic images of RNA ISH against *Pomc*, *Glp1r* and *Lepr* in C57BL/6N mice at 12 weeks of age. First image shows ISH in the ARC with nuclear counterstain (blue, DAPI). Magnifications of the dashed box (right) are shown with the indicated stainings. *Pomc*-positive neurons are outlined in white. Yellow and cyan arrows indicate *Lepr*-positive or *Glp1r*-positive POMC neurons, respectively. Scale bars represent 100 μm in the merged image and 25 μm in the magnifications. **b**, Percentage of *Pomc*-positive cells expressing *Lepr*, *Glp1r* or both receptors across the rostrocaudal axis. The bar graph on the right depicts the total percentage of POMC neurons coexpressing the receptors as averaged from the individual areas. Left: *Lepr*^+^_Rostral_: 22.18% ± 5.02%, *Lepr*^+^_Mid_: 27.54% ± 4.98%, *Lepr*^+^_Caudal_: 24.21% ± 2.49%; *Glp1r*^+^_Rostral_: 27.44% ± 4.73%, *Glp1r*^+^_Mid_: 44.16% ± 3.48%, *Glp1r*^+^_Caudal_: 32.73% ± 11.15%; *Lepr*^+^/*Glp1r*^+^_Rostral_: 8.09% ± 2.44%, *Lepr*^+^/*Glp1r*^+^_Mid_: 12.79% ± 3.08%, *Lepr*^+^/*Glp1r*^+^_Caudal_: 9.40% ± 3.96%. Right: *Lepr*^+^, 24.32% ± 3.31%; *Glp1r*^+^, 35.03% ± 3.89%; *Glp1r*^*+*^/*Lepr*^+^, 10.17% ± 2.10%. One-way ANOVA, *F* (1.656, 4.968) = 64.61, *P* = 0.0003, followed by Tukey’s post hoc test; *Glp1r*^+^ versus *Lepr*^+^ *P* = 0.0536, *Glp1r*^*+*^ versus *Glp1r*^+^/*Lepr*^+^ *P* = 0.0017, *Lepr*^+^ versus *Glp1r*^+^/*Lepr*^+^ *P* = 0.0129; *n* = 4 mice. *P* values were calculated on the total percentage of subpopulations using one-way repeated-measures ANOVA followed by Tukey’s test. *P ≤ 0.05, **P ≤ 0.01, ***P ≤ 0.001. **c**, Illustrations of experimental mice and schematic diagram showing Dre- and Cre-dependent recombination of ROSA26lSlrSrZsGreen reporter line. Excision of loxP- or rox-flanked stop cassettes through recombination of both Dre and Cre drivers led to ZsGreen expression in the targeted POMC population. **d**, Representative microscopic images of immunohistochemical staining against POMC and ZsGreen in the ARC of all resulting genotypes at 15 weeks of age. Scale bar, 50 μm. **e**, Percentage of ZsGreen-positive cells coexpressing or lacking expression of *Pomc*, quantified from RNA ISH. POMC^Dre^ Lepr^Cre^: *Pomc*, 99.21% ± 0.79%; non-*Pomc*, 0.79% ± 0.79%; POMC^Dre^ Glp1r^Cre^: *Pomc*, 94.56% ± 3.43%; non-*Pomc*; 5.44% ± 0.3.43%; *n* = 3 mice per group; minimum of eight sections analyzed for each. For **d** and **e**, data are presented as mean ± s.e.m.

Leptin and Glp1 target POMC neurons to mediate at least part of their metabolism-regulatory functions^4,19^. Single-cell sequencing had revealed no overlap between *Glp1r* and *Lepr* expression in POMC neurons^12^. We therefore further examined whether *Lepr* and *Glp1r* mRNAs were indeed not coexpressed in POMC neurons. We used double fluorescence RNA ISH against *Lepr* and *Glp1r* expression in POMC neurons of wild-type mice, which revealed that while 10.2% of POMC neurons expressed mRNAs of both receptors, the larger proportion of POMC neurons expressed one receptor in the absence of the other (Fig. 2a,b).

**Extended Data Fig. 2:**
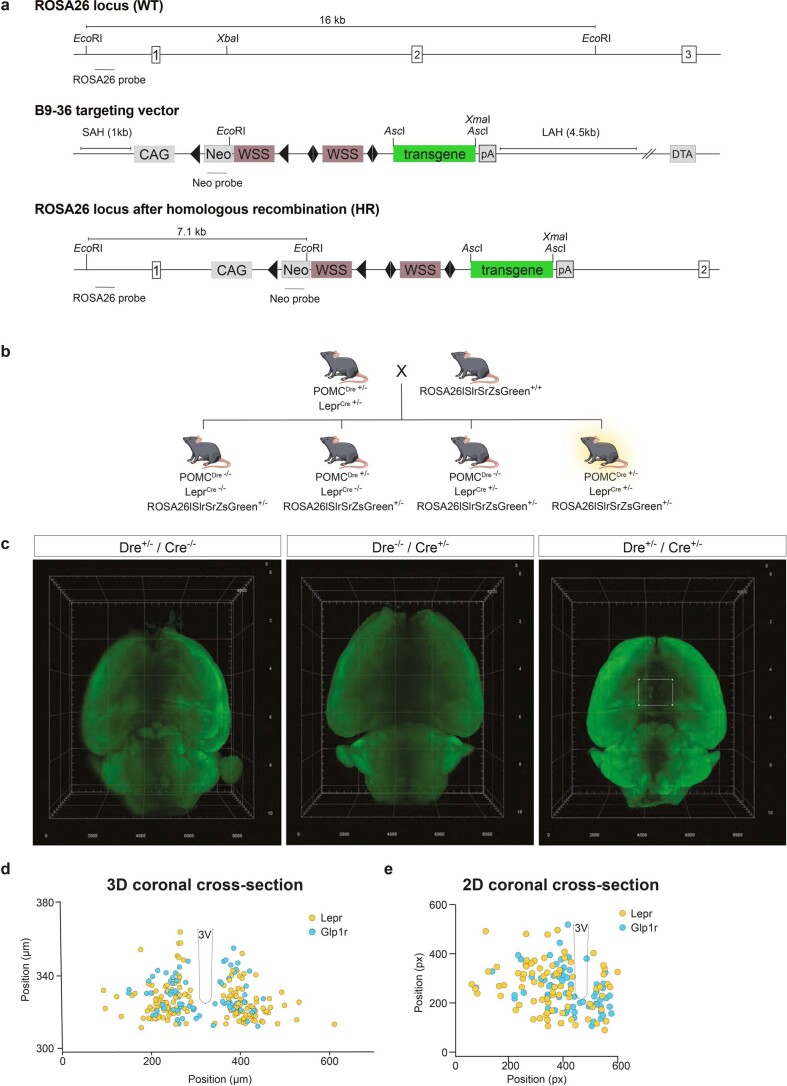
Schematic diagram of the B9-36 targeting, breeding scheme for experimental mice, whole brain images displaying ZsGreen expression and the comparison of coronal sections in 2D vs 3D. **a**, Schematic diagram of the B9-36 targeting and Southern blot strategy for transgene expression dependent on intersectional Cre- and Dre-recombinase activity. Filled triangles: loxP sites; Filled diamonds: rox sites; SAH: short arm of homology; LAH: long arm of homology; WSS: Westphal Stop Sequence; NEO: neomycin resistance gene; CAG: chicken-β-actin promotor; WPRE: woodchuck hepatitis post-transcriptional regulatory element; 2 A: self-cleaving T2A peptide sequence. **b**, Breeding scheme for obtaining experimental mice, depicting the triple transgenic mice and their littermate controls. **c**, Left to right: Whole brain imaging of Pomc^Dre+^ Lepr^Cre−^ ROSA26rSrlSlZSGreen^+/−^, Pomc^Dre−^ GLP1R^Cre+^ ROSA26rSrlSlZSGreen^+/−^, Pomc^Dre+^ Lepr^Cre+^ ROSA26rSrlSlZSGreen^+/−^ 15-week-old mice, respectively. ZsGreen signal specific to the POMC^lepr+^ in the ARC is displayed in the boxed area within the last image on the right. No ectopic expression was observed in only-Cre+ or only-Dre+ mice as depicted in the images on the left. Whole-brain scans were acquired using the LSFM with total magnification of 1.6X. **d**, Bilateral, coronal view of the distribution pattern of POMC^Lepr+^ and POMC^Glp1r+^ neurons in POMC^Dre^ Lepr^Cre^ ROSA26lSlrSrZsGreen and POMC^Dre^ Glp1r^Cre^ ROSA26lSlrSrZsGreen mice. Coronal cross-sections were generated by extracting the 3D coordinates of transgenically labelled POMC^Lepr+^ and POMC^Glp1r+^ neurons in the given section. Data was merged from n = 3 mice/group. **e**, Unilateral, coronal view of the distribution pattern of POMC^Lepr+^ and POMC^Glp1r+^ neurons labelled via RNA *in situ* hybridization. Data was merged from n = 3 mice/group.

To genetically mark heterogeneous POMC cell populations through Cre/Dre-dependent, intersectional recombination, we established reporter mouse models carrying a cDNA encoding the fluorescent marker protein NLS-ZsGreen in the ROSA26 locus^20^. The expression of ZsGreen is prevented by a loxP-flanked (lSl) and an additional rox-flanked (rSr) transcriptional STOP cassette (Fig. 2c). POMC^Dre^ mice were crossed to Lepr^Cre^ or Glp1r^Cre^ mice^21^. Resulting double transgenic POMC^Dre^ Lepr^Cre^ or POMC^Dre^ Glp1r^Cre^ mice were bred with homozygous ROSA26lSlrSrZsGreen^+/+^ mice to yield four different genotypes. This produced mice heterozygous for the reporter (ROSA26lSlrSrZsGreen^+/−^) in the absence of POMC^Dre^ and of the respective Cre transgene, carrying only the POMC^Dre^ transgene, carrying only the respective Cre transgene or the combination of both; that is, POMC^Dre^ Lepr^Cre^ or POMC^Dre^ Glp1r^Cre^ (Extended Data Fig. 2b). Assessment of ZsGreen expression in these mice revealed that ZsGreen immunofluorescence was absent in the ARC of mice carrying neither recombinase transgene or which only carried the POMC^Dre^ or the respective Cre transgene (Lepr^Cre^ or Glp1r^Cre^; Fig. 2d). In contrast, only triple transgenic mice exhibited ZsGreen expression in the ARC (Fig. 2d). Quantification of ZsGreen-positive neurons expressing *Pomc* revealed that 99.2% of ZsGreen-labeled cells in POMC^Dre^ Lepr^Cre^ mice, and 94.6% of ZsGreen-labeled cells in POMC^Dre^ Glp1r^Cre^ mice expressed *Pomc* (Fig. 2e). Further, light-sheet fluorescence microscopy (LSFM) on cleared brain tissue revealed exclusive expression of ZsGreen-positive cells in the ARC of POMC^Dre^ Lepr^Cre^ ROSA26lSlrSrZsGreen^+/–^ mice (Extended Data Fig. 2c), indicating successful intersectional transgenic marking of selective POMC neuron subpopulations.

### Distinct anatomical distribution of POMC^Lepr+^ and POMC^Glp1r+^ neurons in the ARC

**Fig. 3:**
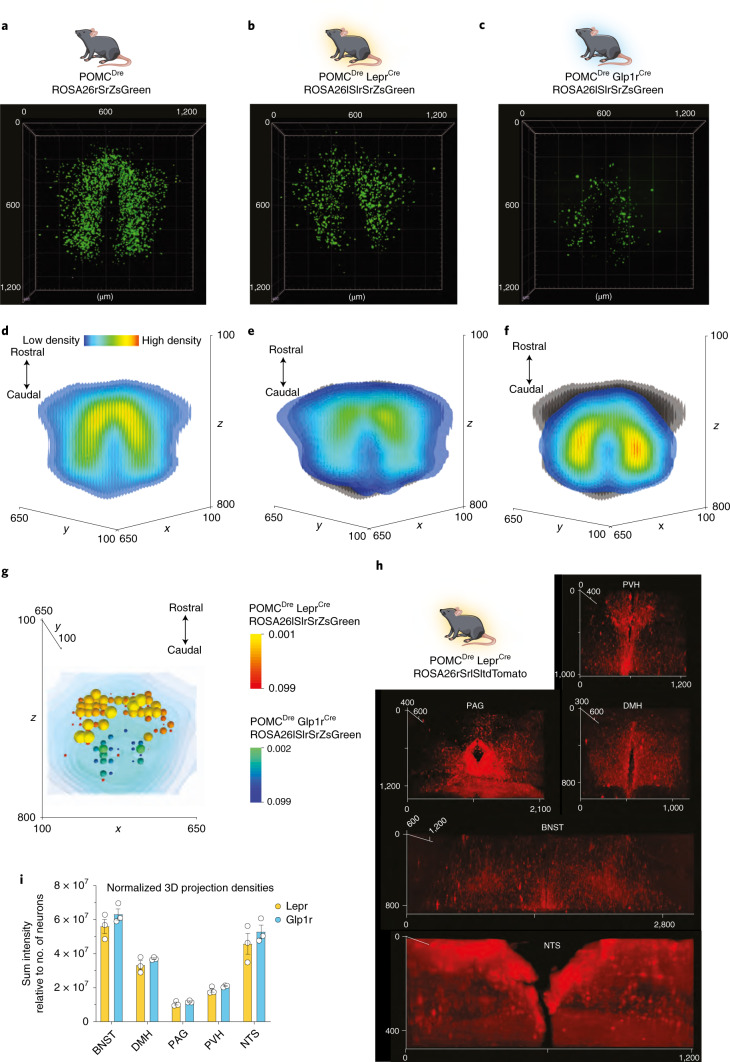
POMC^Lepr+^ and POMC^Glp1r+^ show distinct spatial distribution throughout the ARC. **a–c**, Representative 3D reconstruction of the entire POMC population labeled in POMC^Dre^ ROSA26rSrZsGreen mice (**a**), and 3D reconstruction of POMC subpopulations in POMC^Dre^ Lepr^Cre^ ROSA26lSlrSrZsGreen (**b**) or POMC^Dre^ Glp1r^Cre^ ROSA26lSlrSrZsGreen (**c**) mice at 15 weeks of age. Scans were obtained using the LSFM at ×8 total magnification. *n* = 9 (**a**), *n* = 7 (**b**) and *n* = 8 (**c**) mice. **d–f**, Isosurface density plots of the entire POMC population (**d**), and the POMC^Lepr+^ (**e**) and POMC^Glp1r+^ (**f**) subpopulations. Gray shaded areas in **e** and **f** depict the entire POMC population. **g**, Statistical representation of the differences in distribution between the POMC^Lepr+^ and POMC^Glp1r+^ subpopulations using a two-tailed *t*-test. *P* values are plotted as spheres within the space occupied by the POMC neurons (background). The size and color of the spheres indicate the significance values in ranges of yellow to red (POMC^Lepr+^) and green to blue (POMC^Glp1r+^). **h**, Representative images of 3D projection densities in POMC^Dre^ Lepr^Cre^ ROSA26rSrlSltdTomato mice in the PVH, PAG, DMH, BNST and NTS. **i**, Quantification of 3D projection densities shown in **h**, normalized to the number of neurons. Data are represented as mean ± s.e.m., from *n* = 3 mice per group. POMC^Lepr+^: BNST: 56,068,002.46 ± 4,157,623.02, DMH: 33,195,450.61 ± 2,618,432.01, PAG: 10,481,156.64 ± 819,984.06, PVH: 18,370,352.69 ± 1149,637.53, NTS: 45,834,835.18 ± 6,188,416.90. POMC^Glp1r+^; BNST: 63,188,837.7 ± 3,276,156.35, DMH: 37,050,145.65 ± 478,713.82, PAG: 11,722,976.37 ± 369,033.55, PVH: 20,851,215.3 ± 337,219.38, NTS: 5,294,0731.54 ± 3,984,858.12. BNST, Glp1r^+^ versus Lepr^+^: *P* = 0.249747, *t* = 1.345, df = 4. DMH, Glp1r^+^ versus Lepr^+^: *P* = 0.632027, *t* = 1.448, df = 4. PAG, Glp1r^+^ versus Lepr^+^: *P* = 0.632027, *t* = 1.381, df = 4. PVH, Glp1r^+^ versus Lepr^+^: *P* = 0.432578, *t* = 2.071, df = 4. NTS, Glp1r^+^ versus Lepr^+^: *P* = 0.632027, *t* = 0.9654, df = 4, unpaired Student’s *t*-test, Holm–Sidak correction.

To obtain a holistic 3D representation of the neuronal subpopulations, we used the tissue-clearing technique uDISCO^22^ in combination with LSFM. The 3D images obtained via LSFM from each individual mouse were registered onto a reference atlas, that is, a grayscale Nissl volume of reconstructed brain (Allen Brain 25-μm reference atlas), for subsequent quantitative image analysis. Image registration algorithms vary based on the transformation models they use to relate the target image space to the reference image space. Thus, we used the VINCI software for this purpose^23^. The neuronal coordinates were subsequently extracted and plotted as an isosurface density plot using a kernel mesh fit onto the neuronal population (Fig. 3a–f). Statistical analysis of these neuronal distributions showed significant differences in the localization patterns of Lepr-expressing POMC neurons in comparison to those with Glp1r expression (Fig. 3g). To compare this distribution pattern of POMC cells endogenously expressing *Lepr* or *Glp1r* as assessed by RNA ISH (Fig. 2a), we created coronal cross sections from the 3D coordinates of transgenically labeled POMC^Lepr+^ and POMC^Glp1r+^ neurons (Extended Data Fig. 2d) and compared them to the distributions of POMC neurons endogenously expressing *Lepr* and *Glp1r* in a corresponding anatomical localization (Extended Data Fig. 2e), revealing a similar, differential distribution pattern of both subpopulations in the two experimental approaches.

### Similar projection patterns of POMC^Lepr+^ and POMC^Glp1r+^ neurons

**Extended Data Fig. 3:**
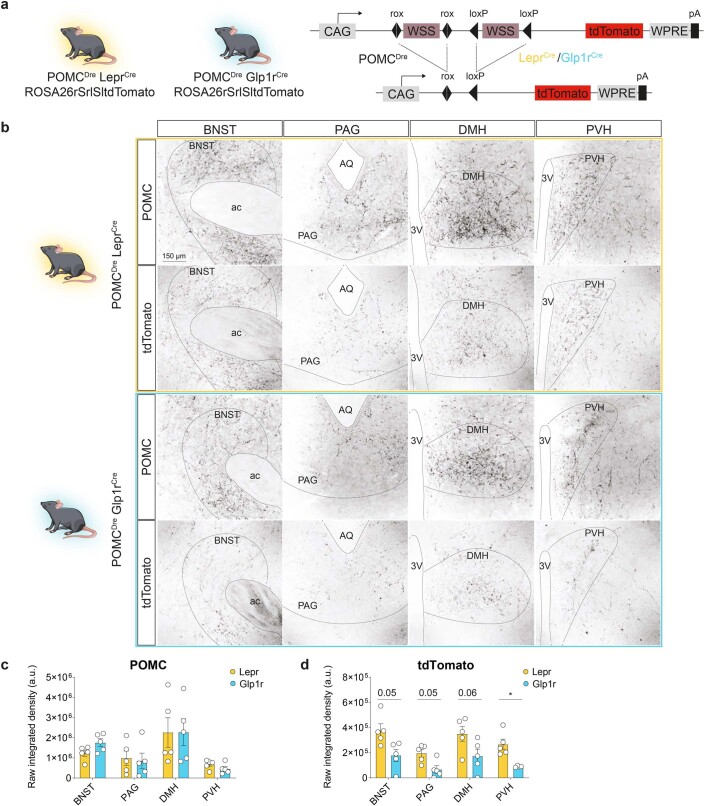
Cre/Dre-dependent tdTomato transgenic lines used for projection analysis of POMCLepr+ and POMCGlp1r+ neurons in 2D sections. **a**, Illustration of experimental mice and schematic diagram showing Dre- and Cre-dependent targeted expression of tdTomato in either POMC^Lepr+^ or POMC^Glp1r+^ neurons. Excision of loxP and rox-flanked stop cassettes through recombination of both Cre and Dre drivers leads to tdTomato expression in the targeted subpopulation. **b**, Representative microscopic images of immunofluorescent staining against tdTomato and POMC in 12-week-old POMC^Dre^ Lepr^Cre^ ROSA26rSrlSltdTomato and POMC^Dre^ Glp1r^Cre^ ROSA26rSrlSltdTomato in the anterior bed nucleus of the stria terminalis (BNST), the periaqueductal gray (PAG), the dorsomedial hypothalamic area (DMH) and the paraventricular nucleus of the hypothalamus (PVH). Scale bar represents 150 μm. 3 V = third ventricle, ac = anterior commissure, AQ = cerebral aqueduct. **c-d**, Quantification of POMC (**c**) and tdTomato fiber density (**d**) assessed as raw integrated density of immunofluorescent staining depicted in (**b**). **c**, n = 5 mice and a minimum of 3, 3, 2, 1section(s) per mouse were analyzed for the BNST, PVH, DMH and PAG. **d**, n = 5 mice and a minimum of 3,3,2,1 sections per mouse were analyzed for the BNST, PVH, DMH and PAG. In **d** and **e**, data are represented as mean ± SEM. *p ≤ 0.05. Further statistical details are given in Source data extended Fig. 2 Source data extended Fig. 2. Source data

To investigate, whether the Lepr- or Glp1r-expressing POMC subpopulations could target distinct projection sites, we used mice expressing tdTomato upon Cre/Dre-dependent recombination (Extended Data Fig. 3a). POMC^Dre^ Lepr^Cre^ ROSA26rSrlSltdTomato^+/−^ and POMC^Dre^ Glp1r^Cre^ ROSA26rSrlSltdTomato^+/−^ animal samples allowed for visualization of axonal projections and dendrites of POMC^Lepr+^ and POMC^Glp1r+^ neurons (Extended Data Fig. 3e)^16^. Sections from well-defined projection areas of melanocortin neurons in the bed nucleus of the striae terminalis (BNST), periaqueductal gray (PAG), dorsomedial nucleus of the hypothalamus (DMH) and the paraventricular nucleus of the hypothalamus (PVH) were examined for immunoreactive fiber density by the quantification of POMC expression and transgenic tdTomato labeling (Extended Data Fig. 3b,c). tdTomato immunoreactive fiber density in the investigated areas was consistently higher in sections of Pomc^Dre^Lepr^Cre^ROSA26rSrlSltdTomato^+/−^ mice compared to those of Pomc^Dre^Glp1r^Cre^ROSA26rSrlSltdTomato^+/−^ animals (Extended Data Fig. 3d), as explained by the larger population of genetically marked POMC^Lepr+^ compared to POMC^Glp1r+^ neurons.

**Extended Data Fig. 4:**
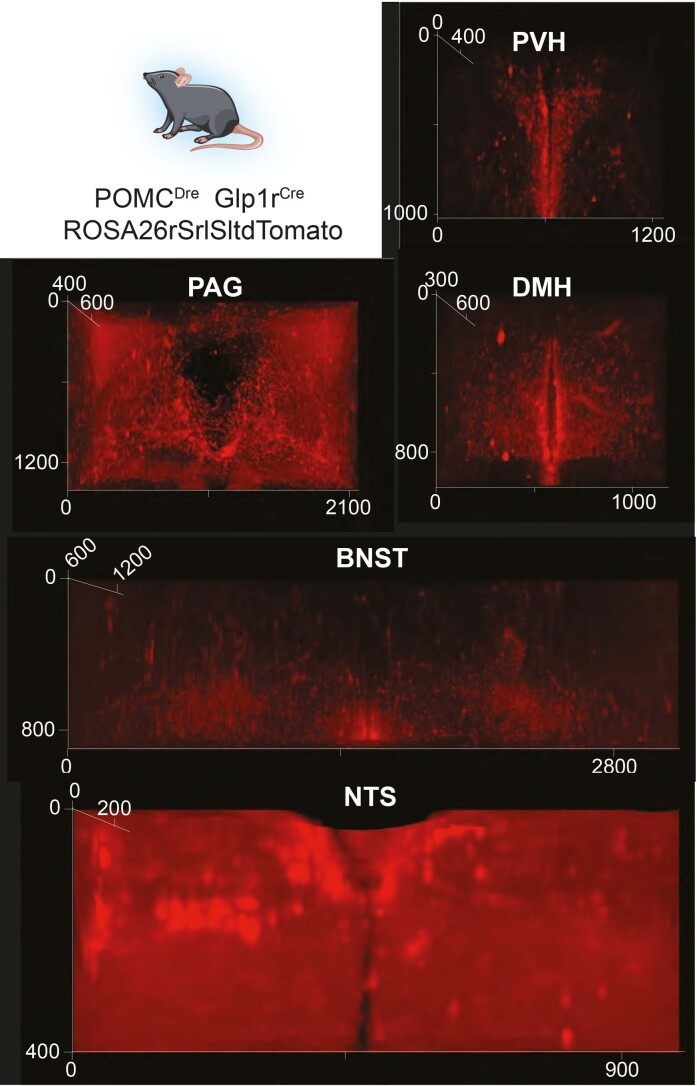
Visualized 3D projection densities of POMC^Glp1r+^ neurons. Representative images of 3D-rendered tdTomato fiber density in POMC^Dre^ Glp1r^Cre^ ROSA26lSlrSrtdTomato mice in the paraventricular nucleus of the hypothalamus (PVH), the periaqueductal gray (PAG), the dorsomedial hypothalamic area (DMH), the anterior bed nucleus of the stria terminalis (BNST) and the nucleus tractus solitarius (NTS).

We also analyzed the projection densities in POMC target regions via whole-brain tdTomato immunostaining and LSFM-based image acquisition followed by data processing based on co-registration onto a unified anatomical atlas. This allowed for assessment of projection densities in the different regions of interest (ROIs; Fig. 3h and Extended Data Fig. 4). At the same time, projection intensities could be normalized to the number of transgenically labeled POMC neurons of the individual animals, revealing a similar projection intensity in the different regions taking into account the differential population sizes of both POMC subpopulations (Fig. 3i).

### DREADD-dependent activation of POMC^Lepr+^ and POMC^Glp1r+^ neurons differentially regulates food intake

**Fig. 4:**
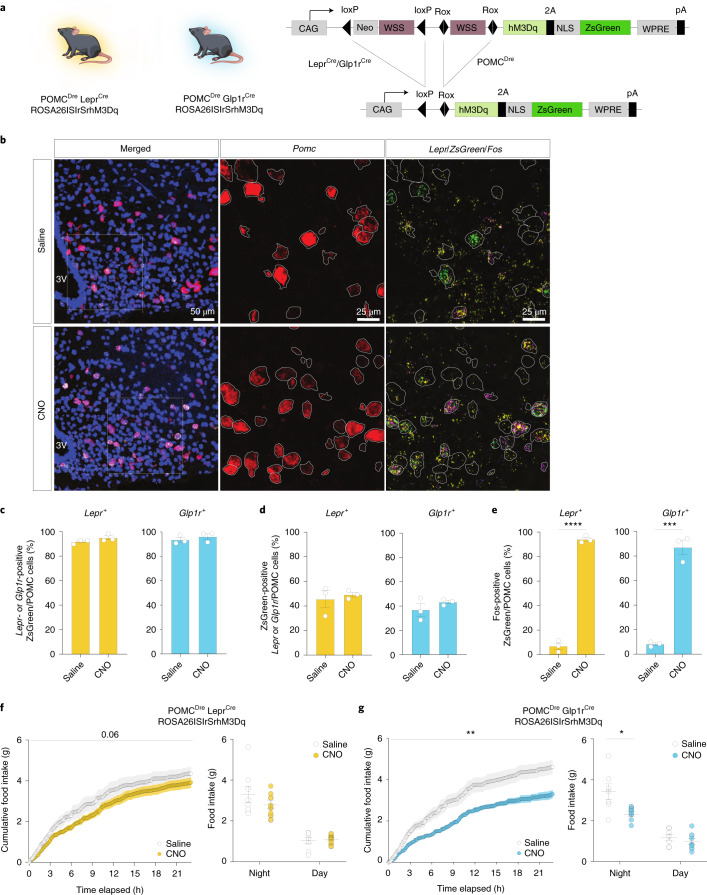
DREADD-dependent activation of POMC^Lepr+^ or POMC^Glp1r+^ neurons differentially reduces food intake. **a**, Illustrations of experimental mice and schematic diagram showing Dre- and Cre-dependent targeted expression of activatory hM3Dq in either POMC^Lepr+^ or POMC^Glp1r+^ neurons. Excision of loxP-flanked and rox-flanked stop cassettes through recombination of both Dre and Cre drivers leads to hM3Dq expression in the targeted subpopulation. **b**, Representative microscopic images of RNA ISH against *Pomc*, *Lepr*, *ZsGreen* (in lieu of hM3Dq) and *Fos* in POMC^Dre^ Lepr^Cre^ ROSA26lSlrSrhM3Dq males injected with saline or CNO. Images on the left show ISH in the ARC with nuclear counterstain (blue, DAPI). Magnifications of the boxes (right) are shown with the indicated stainings. *Pomc*-positive neurons are outlined in white. Scale bars, 50 μm (merged image) and 25 μm (magnified images). **c–e**, Percentage of ZsGreen-*Pomc*-positive cells expressing *Lepr* or *Glp1r* (**c**), percentage of *Lepr/Glp1r*-*Pomc*-positive cells expressing ZsGreen (**d**) and percentage of ZsGreen-*Pomc*-positive cells expressing *Fos* (**e**) in POMC^Dre^ Lepr^Cre^ ROSA26lSlrSrhM3Dq or POMC^Dre^ Glp1r^Cre^ ROSA26lSlrSrhM3Dq male mice (22–26 weeks old) injected with saline or CNO. CNO, 3 mg kg^−1^. **c**: POMC^Lepr+^: saline: 91.84% ± 1.03%, CNO: 94.87% ± 1.62%, saline versus CNO, *t* = 1.580, *P* = 0.342863: POMC^Glp1r+^: saline: 93.52% ± 2.04%, CNO: 96.16% ± 2.31% saline versus CNO, *t* = 0.8592, *P* = 0.438695; **d**: POMC^Lepr+^: saline: 45.46% ± 6.92%, CNO: 49.19% ± 1.92%, saline versus CNO, *t* = 0.5191, *P* = 0.631109; POMC^Glp1r+^: saline: 36.88% ± 5.28%, CNO: 43.24% ± 1.42%, saline versus CNO, *t* = 1.165, *P* = 0.522274. **e**: POMC^Lepr+^: saline: 6.87% ± 2.64%, CNO: 94.03% ± 1.66%, saline versus CNO, t = 28.0, df = 4, *P*_*uT*_ = 0.000019; POMC^Glp1r+^: saline: 8.23% ± 1.13%, CNO: 87.05% ± 6.05%, saline versus CNO, *t* = 12.82, df = 4, *P*_*uT*_ = 0.000214, unpaired Student’s *t*-test, Holm–Sidak correction; *n* = 3 mice. **f**,**g**, Food intake over a time course of 24 h in POMC^Dre^ Lepr^Cre^ ROSA26lSlrSrhM3Dq (**f**) and POMC^Dre^ Glp1r^Cre^ ROSA26lSlrSrhM3Dq male mice (**g**) starting with the night cycle. Mice were injected with saline at 18:00 and 23:00, followed by a 1-d gap and subsequent CNO injections at 18:00 and 23:00 on the next day. Left: cumulative food intake in mice injected with saline versus CNO; right, total food intake in grams during night and day. **f:** *n* = 8; left: saline versus CNO two-way ANOVA, *F*(1,7) = 4.815, *P* = 0.0643; right: saline_Night_: 3.03 ± 0.39, CNO_Night_: 2.79 ± 0.20, saline versus CNO two-way ANOVA followed by Sidak’s test, *P* = 0.2729, saline_Day_: 1.05 ± 0.15, CNO_Day_: 1.10 ± 0.07, saline versus CNO two-way ANOVA followed by Sidak’s test, *P* = 0.9867. **g**: *n* = 7, left: saline versus CNO two-way ANOVA, *F*(1.000, 6.000) = 16.51, *P* = 0.0066, right: saline_Night_: 3.42 ± 0.38, CNO_Night_: 2.32 ± 0.12, saline_Day_: 1.19 ± 0.15, CNO_Day_: 0.98 ± 0.16; saline versus CNO two-way ANOVA followed by Sidak’s test, *P* = 0.0312; saline versus CNO two-way ANOVA followed by Sidak’s test, *P* = 0.7992. Data are represented as the mean ± s.e.m. Statistical analyses in **c**–**e** were performed by unpaired two-tailed Student’s *t*-test with Holm–Sidak correction for multiple comparisons. For cumulative food intake (**f** and **g** left), two-way ANOVA was used; for total food intake (**f** and **g** right), two-way ANOVA followed by Sidak’s post hoc test was used. Indices *P*_uT_: unpaired *t*-test. *P ≤ 0.05, **P ≤ 0.01, ***P ≤ 0.001, ***P ≤ 0.0001.

**Extended Data Fig. 5:**
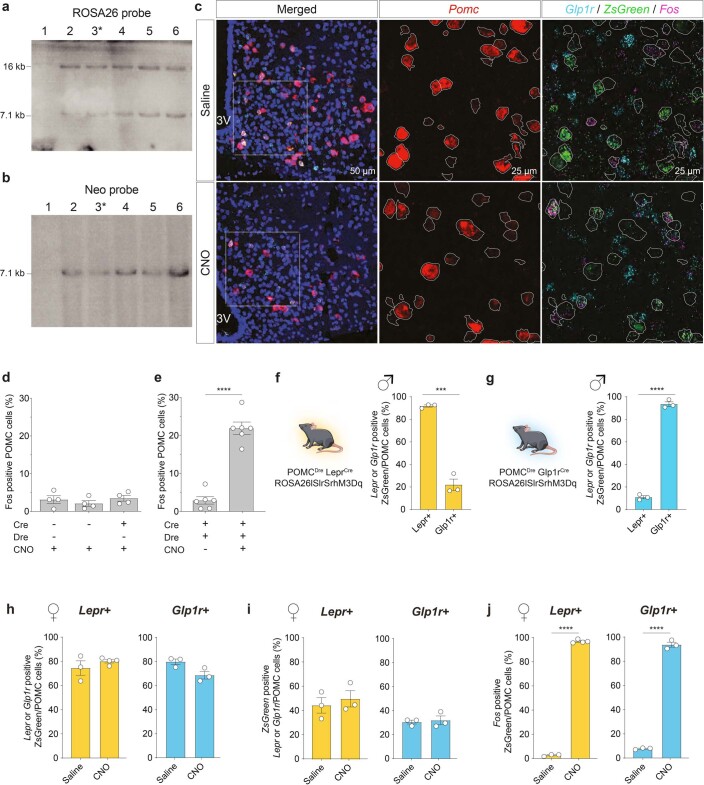
Cre/Dre-dependent hM3Dq transgenic lines allow for specific neuronal activation solely in triple transgenic mice. **a-b**, Southern blots of R26lSlrSrhM3Dq mouse line with the ROSA26 (**a**) and NEO probe (**b**). The asterisk indicates the clone chosen for blastocyst injection. (**c**) Representative microscopic images of RNA *in situ* hybridization against *Pomc*, *Glp1r, ZsGreen* (in lieu of hM3Dq) and *Fos* in POMC^Dre^ Glp1r^Cre^ ROSA26lSlrSrhM3Dq mice injected with saline (top) or CNO (bottom). Images on the left show ISH in the ARC with nuclear counterstain (blue, DAPI). Magnifications of the dashed boxes are displayed on the right showing the indicated stainings. *Pomc*-positive neurons have been traced with a white outline. Scale bars represent 50 μm in the merged image and 25 μm in the magnifications. 3 V = third ventricle. **d-e**, Percentage of *Pomc*-positive cells expressing *Fos* in the different CNO-injected genotype controls (**d**) and triple transgenic animals injected with saline or CNO (**e**) of POMC^Dre^ Lepr^Cre^ ROSA26lSlrSrhM3Dq and POMC^Dre^ Glp1r^Cre^ ROSA26lSlrSrhM3Dq mice. **d**, n = 4 mice, **e**, n = 5 and 6 mice for groups with and without CNO, respectively. **f-g**, Percentage of Lepr^+^ or Glp1r^+^ cells in (**f**) POMC^Dre^ Lepr^Cre^ ROSA26lSlrSrhM3Dq and (**g**) POMC^Dre^ Glp1r^Cre^ ROSA26lSlrSrhM3Dq male mice as quantified from RNA *in situ* hybridization. n = 3 mice. **h-j**, Percentage of *ZsGreen*-*Pomc*-positive cells expressing *Lepr* or *Glp1r* (**h**), percentage of *Lepr/Glp1r*-*Pomc*-positive cells expressing *ZsGreen* (**i**) and percentage of *ZsGreen*-*Pomc*-positive cells expressing *Fos* (**j**) in POMC^Dre^ Lepr^Cre^ ROSA26lSlrSrhM3Dq or POMC^Dre^ Glp1r^Cre^ ROSA26lSlrSrhM3Dq females (22–26 weeks old) injected with saline or CNO. **j**; POMC^Lepr+^, saline n = 3, CNO n = 4, saline vs CNO *p* < 0.000001, POMC^Glp1r+^, saline n = 3, CNO n = 3, saline vs CNO *p* = 0.000002. n indicates number of mice. Data are represented as mean ± SEM. CNO = 3 mg/kg. Statistical analyses on (**b**) were assessed by two-way-ANOVA. All other statistical analyses were performed by unpaired two-tailed Student’s t-test with (**h**-**j**) or without (**e**-**g**) Holm-Sidak correction for multiple comparisons. ***p ≤ 0.001, ****p ≤ 0.0001. Further statistical details are given in Source data extended Fig. 3 Source data extended Fig. 3. Source data

To investigate the role of distinct POMC neuron populations in energy homeostasis in vivo, we generated mice allowing for combinatorial Cre/Dre-dependent expression of the activatory DREADD (Designer Receptors Exclusively Activated by Designer Drugs) receptor hM3Dq and ZsGreen^14^ (Fig. 4a and Extended Data Fig. 5a,b). Male POMC^Dre^Lepr^Cre^ROSA26lSlrSrhM3Dq^+/−^ animals were injected intraperitoneally (i.p.) with either saline or clozapine *N*-oxide (CNO). Activation of POMC^Lepr+^ neurons was assessed via RNAscope ISH using probes against *Pomc*, *Lepr*, *ZsGreen* and *Fos* (Fig. 4b). The majority of ZsGreen-expressing POMC neurons were positive for *Lepr* expression (93.4%), supporting specific targeting of POMC^Lepr+^ neurons (Fig. 4c). Furthermore, the proportion of ZsGreen-expressing cells that coexpressed *Pomc* and *Lepr* mRNA was assessed, revealing the expression of ZsGreen (and thus hM3Dq) in 47.3% of the cells expressing *Lepr* and *Pomc* (Fig. 4d). To investigate the efficiency of CNO-dependent cell activation, the ratio of *Fos*-positive cells over ZsGreen and *Pomc* double-positive neurons was assessed in saline-injected and CNO-injected POMC^Dre^ Lepr^Cre^ ROSA26lSlrSrhM3Dq^+/−^ animals. While only 6.9% of hM3Dq-expressing POMC neurons were positive for *Fos* mRNA expression in saline-injected animals, this proportion increased to 94.0% after CNO injection (Fig. 4e). Similarly, POMC^Dre^ Glp1r^Cre^ ROSA26lSlrSrhM3Dq^+/−^ animals exhibited CNO-dependent activation of POMC^Glp1r+^ neurons in males (Fig. 4c–e and Extended Data Fig. 5c). Moreover, CNO-only injection elicited increased *Fos* expression in POMC neurons of POMC^Dre^ Lepr^Cre^ ROSA26lSlrSrhM3Dq^+/−^ and POMCDre Glp1r^Cre^ ROSA26lSlrSrhM3Dq^+/−^ animals but not in ROSA26lSlrSrhM3Dq^+/−^animals lacking Dre and Cre expression or expressing either recombinase alone (Extended Data Fig. 5d,e). Moreover, more than 90% of the labeled POMC neurons in POMC^Dre^ Lepr^Cre^ ROSA26lSlrSrhM3Dq^+/−^ mice expressed *Lepr*, while 22.2% expressed *Glp1r*. In turn, only 10.9% of the labeled neurons in POMC^Dre^ Glp1r^Cre^ ROSA26lSlrSrhM3Dq^+/−^ mice were Lepr positive and 93.5% expressed Glp1r (Extended Data Fig. 5f,g). Finally, CNO application similarly activated POMC^Lepr+^ and POMC^Glp1r+^ neurons in female triple transgenic animals as observed in males (Extended Data Fig. 5h–j).

**Extended Data Fig. 6:**
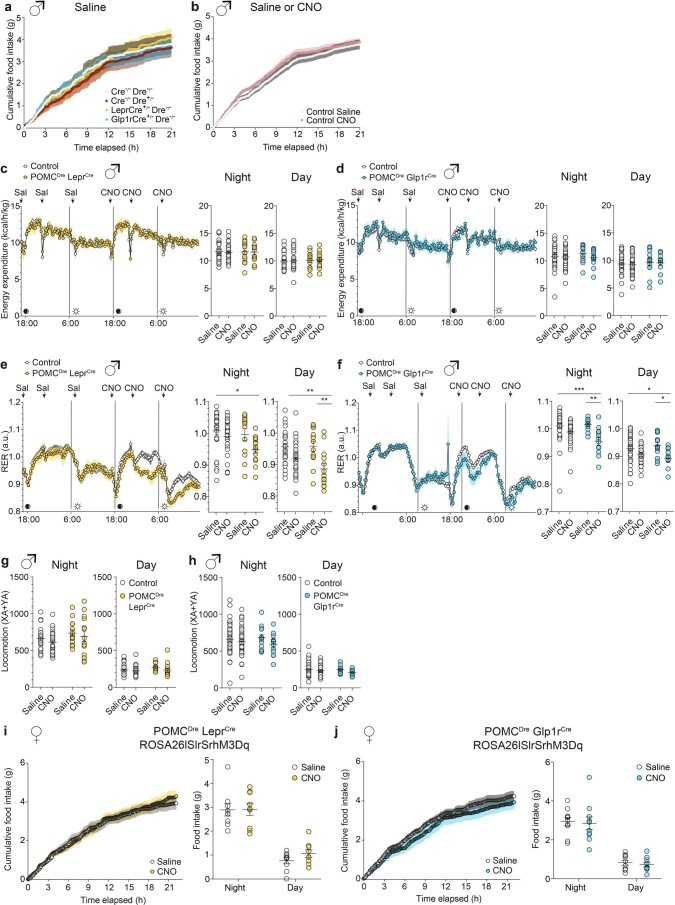
Metabolic phenotyping of Cre/Dre-dependent ROSA26lSlrSrhM3Dq in males and food intake measurements in female mice. **a-b**, Cumulative food intake over a time course of 24 hours in all saline-injected genotype controls (**a**) and pooled saline- or CNO-injected control animals vs. saline-injected POMC^Dre^ Lepr^Cre^ ROSA26lSlrSrhM3Dq and POMC^Dre^ Glp1r^Cre^ ROSA26lSlrSrhM3Dq male mice (**b**) starting with the night cycle. Mice were injected with saline at 18:00 and 23:00, followed by one-day gap and subsequent CNO injections at 18:00 and 23:00 on the next day. Data of all genotype controls in (**a**) is shown as pooled saline-injected Control in (**b**), data of saline-injected triple transgenic animals (**a**) are shown in Fig. 4f and g as control group. (**a**) Cre-/Dre-, Cre-/Dre+, LeprCre+/Dre-, Glp1rCre+/Dre-, n = 8, 9, 4 and 5 mice respectively. (**b**) Control Saline n = 25 and Control CNO n = 26 mice. **c-h**, Energy expenditure (**c**,**d**), RER (**e**,**f**) and locomotion (**g**,**h**) in POMC^Dre^ Lepr^Cre^ ROSA26lSlrSrhM3Dq (**c**,**e**,**g**) and POMC^Dre^ Glp1r^Cre^ ROSA26lSlrSrhM3Dq male mice (**d**,**f**,**h**) vs. controls at 12–14 weeks of age. Mice were injected with saline at 17:00, 22:00 and 07:00 followed by CNO at 17:00, 22:00 and 07:00 on the next day. Scatter plots on the right show the average values during night and day as averaged from left graph. Values of control animals were pooled from all corresponding genotype controls. Sal=Saline. **c** and **e**, Control n = 29, POMC^Dre^ Lepr^Cre^ n = 13. **d** and **f**, Control n = 38, POMC^Dre^ Glp1r^Cre^ n = 12. **g**, Control n = 31, POMC^Dre^ Lepr^Cre^ n = 14. **h** Control n = 41, POMC^Dre^ Glp1r^Cre^ n = 12. E_right_; Night: Control_Saline_ vs POMC^Lepr+^_CNO_, *p* = 0.016, Day: Control_Saline_ vs POMC^Lepr+^_CNO_, *p* = 0.0014, POMC^Lepr+^_Saline_ vs POMC^Lepr+^_CNO_, *p* = 0.0065, Day: Control_Saline_ vs POMC^Lepr+^_CNO_, *p* = 0.001. F_right_; Night: Control_Saline_ vs POMC^Glp1r+^_CNO_, *p* = 0.001, Control_CNO_ vs POMC^Glp1r+^_CNO_, *p* = 0.0049, Day: Control_Saline_ vs POMC^Glp1r+^_CNO_, *p* = 0.035, POMC^Glp1r+^_Saline_ vs POMC^Glp1r+^_CNO_, *p* = 0.028. **i-j**, Food intake over a time course of 24 hours in POMC^Dre^ Lepr^Cre^ ROSA26lSlrSrhM3Dq (**i**) and POMC^Dre^ Glp1r^Cre^ ROSA26lSlrSrhM3Dq female mice (**j**) starting with the night cycle. Mice were injected with saline at 18:00 and 23:00, followed by one day gap and subsequent CNO injections at 18:00 and 23:00 on the next day. Left: Cumulative food intake in mice injected with saline vs. CNO. Right: Total food intake during night and day. n = 9 mice/group (**i**), n = 10 mice/group (**j**). CNO = 3 mg/kg. Data are represented as mean ± SEM. All *p*-values were calculated by two-way-ANOVA. For cumulative food intake (**i**-**j**, left) two-way-ANOVA was used, for total food intake (**i**-**j**, right) two-way-ANOVA followed by Sidak’s post-hoc test. In **c**-**h** significance was calculated exclusively on scatter plots and ANOVA was followed by Tukey’s post-hoc test, where relevant. *p ≤ 0.05, **p ≤ 0.01, ***p ≤ 0.001. Further statistical details are given in Source data extended Fig. 4 Source data extended Fig. 4. Source data

Next, we assessed parameters of energy homeostasis upon treating POMC^Dre^ Lepr^Cre^ ROSA26lSlrSrhM3Dq^+/−^ or POMC^Dre^ Glp1r^Cre^ ROSA26lSlrSrhM3Dq^+/−^ animals and their respective controls with either saline or CNO, while performing an indirect calorimetry combined with continuous monitoring of food intake. Upon saline treatment feeding rates in male mice of the different genotypes did not differ (Extended Data Fig. 6a). Moreover, repeated injections of CNO in control animals did not affect food intake compared to saline-injected control animals (Extended Data Fig. 6b). In contrast, CNO injections of male POMC^Dre^ Lepr^Cre^ ROSA26lSlrSrhM3Dq^+/−^ mice at the beginning of the natural feeding cycle, reduced food intake starting at 3 h after CNO injection (Fig. 4f). This effect cumulated into a statistically nonsignificant 15.6% suppression of feeding over the duration of the dark cycle (Fig. 4f). However, chemogenetic activation of male POMC^Dre^ Glp1r^Cre^ ROSA26lSlrSrhM3Dq^+/−^ mice resulted in an earlier (2.5 h after CNO injection) and even stronger feeding suppression, accounting for a significant 32.3% reduction in food intake over the duration of the dark cycle (Fig. 4g). In contrast, chemogenetic activation of POMC^Lepr+^ or POMC^Glp1r+^ neurons in male mice did not affect energy expenditure or locomotor activity, while mildly shifting the respiratory exchange ratio toward fatty acid metabolism (Extended Data Fig. 6c–h). Interestingly, chemogenetic activation of POMC^Lepr+^ and POMC^Glp1r+^ neurons in female mice did not suppress feeding.

**Extended Data Fig. 7:**
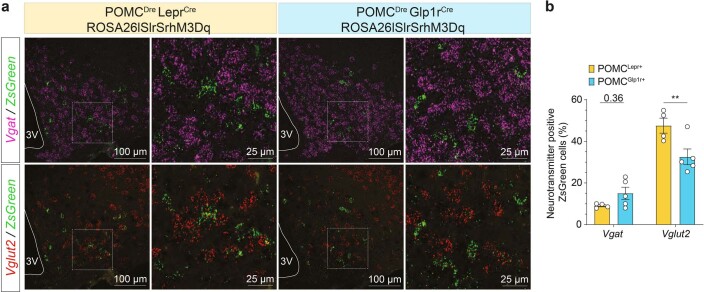
*Vgat* and *Vglut2* expression in POMC^Lepr+^ and POMC^Glp1r+^ neurons. **a**, Representative microscopic images of RNA *in situ* hybridization against *ZsGreen* and *Vgat* or *Vglut2* in POMC^Dre^ Lepr^Cre^ ROSA26lSlrSrhM3Dq (left) and POMC^Dre^ Glp1r^Cre^ ROSA26lSlrSrhM3Dq (right) male mice. Scale bars represent 100 μm in the merged image and 25 μm in the magnifications. **b**, Quantification of the percentage overlap of *Vgat* or *Vglut2* with *ZsGreen*-expressing cells. For POMC^Lepr+^ and POMC^Glp1r+^ n = 4 and 5 mice, respectively. Data are represented as mean ± SEM. Statistical analyses on B were performed by two-way ANOVA with Sidak’s post-hoc test. **p ≤ 0.01. Further statistical details are given in Source data extended Fig. 5. Source data extended Fig. 5 Source data.

Given the different kinetics in feeding suppression upon activation of POMC^Glp1r+^ neurons compared POMC^Lepr+^ neurons in male mice, we asked whether both neuronal subpopulations might differ in their neurotransmitter characteristics. Therefore, we compared the expression of the vesicular GABA transporter (*Vgat* or *Slc32a1*) and of the vesicular glutamate transporter (*Vglut2* or *Slc17a6*) in POMC^Lepr+^ and POMC^Glp1r+^ neurons (Extended Data Fig. 7a). Quantification of the proportion of GABAergic and glutamatergic POMC neurons in both mouse lines revealed a slightly higher proportion of glutamatergic POMC^Lepr+^ neurons. However, non-POMC neurons appeared to exhibit a stronger signal for *Vglut2* expression compared to POMC neurons (Extended Data Fig. 7b).

**Extended Data Fig. 8:**
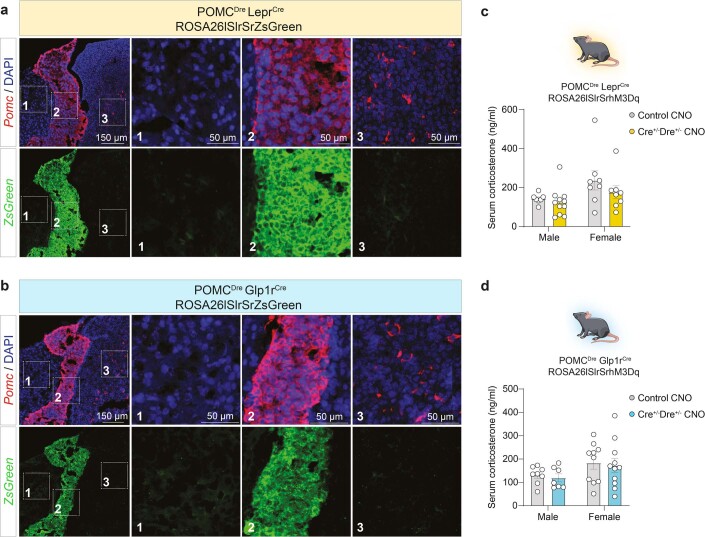
*ZsGreen* expression in pituitary of POMC^Dre^ Lepr^Cre^ ROSA26lSlrSrZsGreen and POMC^Dre^ Glp1r^Cre^ ROSA26lSlrSrZsGreen mice. **a,b**, Representative microscopic images of RNA *in situ* hybridization against *ZsGreen* in pituitary of POMC^Dre^ Lepr^Cre^ ROSA26lSlrSrZsGreen (**a**) and POMC^Dre^ Glp1r^Cre^ ROSA26lSlrSrZsGreen (**b**) mice, respectively. Scale bars represent 150 μm in the merged image and 50 μm in the magnifications. **c,d**, Corticosterone levels in sera of CNO treated POMC^Dre^ Lepr^Cre^ ROSA26lSlrSrhM3Dq (**c**) and POMC^Dre^ Glp1r^Cre^ ROSA26lSlrSrhM3Dq (**d**) male and female mice. Mice were fasted for 2 hours and injected with CNO 1 hour into the fast prior to serum collection. CNO = 3 mg/kg. C males; Controls n = 7, Cre^+^Dre^+^ n = 11, C females; Controls n = 8, Cre^+^Dre^+^ n = 8. D males; Controls n = 8, Cre^+^Dre^+^ n = 7, D females; Controls n = 10, Cre^+^Dre^+^ n = 11. Data are represented as mean ± SEM. Statistical analyses on (**c**-**d**) were performed by unpaired two-tailed Student’s t test with Holm-Sidak correction for multiple comparisons. Further statistical details are given in Source data extended Fig. 6 Source data extended Fig. 6.

Since POMC is also expressed not only in the ARC, but also in the anterior and intermediate lobe of the pituitary, we compared the detectability of *ZsGreen*-positive cells in the pituitary of POMC^Dre^ Lepr^Cre^ ROSA26lSlrSrzsGreen^+/−^ or POMC^Dre^ Glp1r^Cre^ ROSA26lSlrSrzsGreen^+/−^ animals (Extended Data Fig. 8a,b). Although *ZsGreen* expression was detectable in the majority of cells in the intermediate lobe of both POMC^Dre^ Lepr^Cre^ ROSA26lSlrSrzsGreen^+/−^ and POMC^Dre^ Glp1r^Cre^ ROSA26lSlrSrzsGreen^+/−^ animals, we did not detect *ZsGreen*-positive cells in the anterior lobe of the pituitary in either mouse line (Extended Data Fig. 8a,b). Activation of each subpopulation did not alter circulating corticosterone concentrations in the serum of either mouse line (Extended Data Fig. 8c,d).

### Distinct translational signatures of POMC^Lepr+^ and POMC^Glp1r+^ neurons

**Fig. 5:**
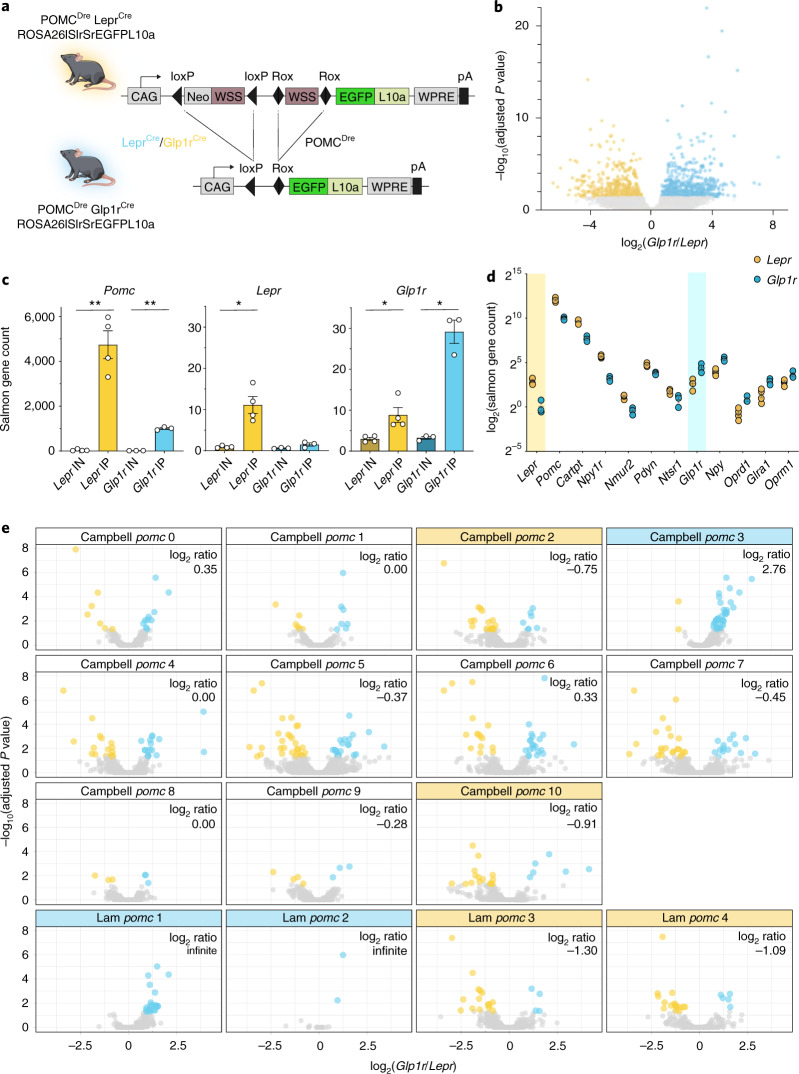
POMC^Lepr+^ and POMC^Glp1r+^ neurons exhibit distinct translational profiles. **a**, Illustration of experimental mice and schematic diagram showing Dre- and Cre-dependent targeted expression of EGFPL10a in either POMC^Lepr+^ or POMC^Glp1r+^ neurons. Excision of *loxP*-flanked and *rox*-flanked stop cassettes through recombination of both Dre and Cre drivers led to EGFPL10a expression in the targeted subpopulation. **b**, Volcano plot of differentially ribotag-enriched transcripts in POMC^Lepr+^ and POMC^Glp1r+^ neurons. Significantly differentially enriched transcripts (*P* ≤ 0.05) are indicated in the colored region. Yellow and cyan depict a significantly higher enrichment in POMC^Lepr+^ and POMC^Glp1r+^ neurons, respectively. *P* values adjusted for multiple comparisons were calculated by DESeq2 (1.26.0). **c**, Expression of *Pomc*, *Lepr and Glp1r* in input (IN) and IP samples of each subpopulation. Statistics were analyzed using unpaired two-tailed Welch’s *t*-test. *Pomc*: *Lepr* IN: 39.44 ± 19.39, *Lepr* IP: 4,740.55 ± 618.41, IN vs IP, *t* = 7.6, *P* = 0.0047. *Glp1r* IN: 14.14.55 ± 0.49, *Glp1r* IP: 1,005.42 ± 40.19, IN vs IP, *t* = 24.7, *P* = 0. 0016. *Lepr*: *Lepr* IN: 0.92 ± 0.14, *Lepr* IP: 11.12 ± 2.05, IN vs IP, *t* = 4.95, *P* = 0.0155. *Glp1r* IN: 0.62 ± 0.07, *Glp1r* IP: 1.50 ± 0.40, IN vs IP, *t* = 2.18, *P* = 0.001536. *Glp1r*: *Lepr* IN: 2.97 ± 0.38, *Lepr* IP: 8.80 ± 1.82, IN vs IP, *t* = 3.15, *P* = 0.0458. *Glp1r* IN: 3.23.55 ± 0.35, *Glp1r* IP: 29.17 ± 2.83, IN vs IP, *t* = 9.08, *P* = 0.0108. *P ≤ 0.05, **P ≤ 0.01. **d**, Significantly differentially enriched genes (*P* ≤ 0.05) of POMC^Lepr+^ and POMC^Glp1r+^ neurons, belonging to the GO term ‘neuropeptide-signaling pathways’. Vertical colored area separates higher enrichment in POMC^Lepr+^ neurons (left) from higher enrichment in POMC^Glp1r+^ neurons (right). **e**, Overlap analysis of the publicly available single-cell RNA-seq data from mouse hypothalami with our dataset. Here, the volcano plot in **b** is filtered for the markers reported for each cluster. For POMC^Dre^ Lepr^Cre^ ROSA26lSlrSrEGFPL10a, *n* = 4 samples of pooled hypothalami from *N* = 24 mice; for POMC^Dre^ Glp1r^Cre^ ROSA26lSlrSrEGFPL10a, *n* = 3 samples of pooled hypothalami from *N* = 36 mice. Data are represented as the mean ± s.e.m.

**Extended Data Fig. 9:**
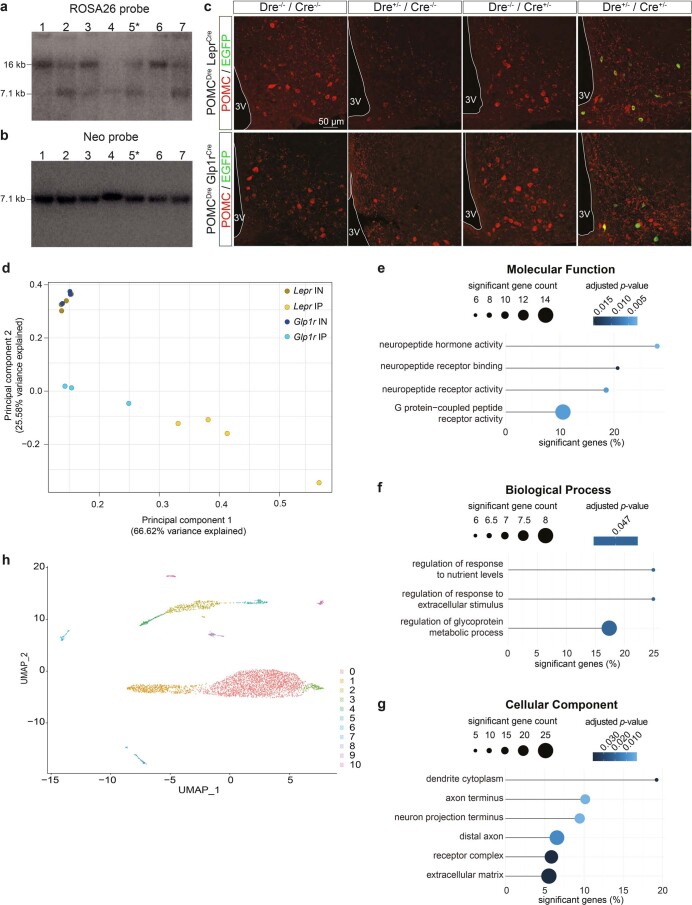
Validation of the Dre-Cre dependent ROSA26lSlrSrEGFPL10a transgenic line. **a-b**, Southern blots of ROSA26lSlrSrEGFPL10a mouse line with the ROSA26 (**a**) and Neo probe (**b**). The asterisk indicates the clone chosen for blastocyst injection. **c**, Representative microscopic images of immunofluorescent staining against POMC and EGFP in the ARC of all resulting genotypes at 12 weeks of age. Scale bar represents 50 μm. 3 V = third ventricle. **d**, Principal component analysis of all RNA input and IP samples. **e-g**, GO term representations of the differentially enriched genes between the POMC^Lepr+^ and POMC^Glp1r+^ clusters mapping the percentage of regulated genes to the total GO term gene count against significant gene count and adjusted *p*-value per term. **h**, UMAP plot of 773 POMC neurons identified in normal chow diet fed mice. Cells that are closer in position in the plot have a similar genetic profile. In (**e**-**g**) *p*-values were calculated using the DESeq2 1.26.0 pipeline with adjustments for multiple comparisons.

To obtain detailed transcriptional/translational profiles of POMC^Lepr+^ and POMC^Glp1r+^ neurons, we generated mice enabling combinatorial Cre/Dre-dependent expression of the L10a ribosomal protein fused to enhanced green fluorescent protein (EGFP; Fig. 5a and Extended Data Fig. 9a,b). Hypothalami of resulting triple transgenic animals (POMC^Dre^ Lepr^Cre^ ROSA26lSlrSrEGFPL10a^+/−^ and POMC^Dre^ Glp1r^Cre^ ROSA26lSlrSrEGFPL10a^+/−^ mice) were used for RNA extraction. Biological replicates for the individual lines provided input RNA samples, which were subjected to anti-EGFP immunopurification of EGFP-tagged ribosomes of POMC^Lepr+^ or POMC^Glp1r+^ neurons. Having confirmed the specific expression of the EGFPL10a fusion protein using the ROSA26lSlrSrEGFPL10a mouse line (Extended Data Fig. 5c) and validated the successful, specific pulldown of RNA of subpopulations of POMC neurons, our aim was to compare the ribosome-associated transcriptome of POMC^Lepr+^ neurons with POMC^Glp1r+^ neurons. Thus, RNA-seq was performed on input and immunoprecipitation (IP) RNA samples of POMC^Dre^ Lepr^Cre^ ROSA26lSlrSrEGFPL10a^+/−^ and POMC^Dre^ Glp1r^Cre^ ROSA26lSlrSrEGFPL10a^+/−^ mice. In total, reads mapped to 17,239 genes. Principal-component analyses for the highest expressed genes showed clear separation of input from IP samples, as well as for POMC^Dre^ Lepr^Cre^ ROSA26lSlrSrEGFPL10a^+/−^ IP from POMC^Dre^ Glp1r^Cre^ ROSA26lSlrSrEGFPL10a^+/−^ IP samples (Extended Data Fig. 9a). Next, the expression level of each IP sample was normalized to its own input sample. The normalized expression analysis of all samples from both triple transgenic mouse groups was used to visualize the differences in expression of all detected genes in both subpopulations. Almost an equal number of genes were expressed at higher levels in the POMC^Lepr+^ compared to POMC^Glp1r+^ neurons and vice versa (Fig. 5b).

As expected, *Pomc* transcript reads were drastically (>1,000-fold) enriched in both POMC^Lepr+^ and POMC^Glp1r+^ neurons compared to the global hypothalamic background (Fig. 5c). POMC^Lepr+^ neurons exhibited a stronger enrichment for *Pomc* mRNA compared POMC^Glp1r+^ neurons, indicating a potentially differential *Pomc* mRNA expression (Fig. 5c). Similarly, we found clear overrepresentation for the reads of *Lepr* in IP samples from POMC^Lepr+^ neurons and an enrichment of *Glp1r* expression in IP samples from POMC^Glp1r+^ neurons (Fig. 5c).

To identify which classes of transcripts differentiate POMC^Lepr+^ and POMC^Glp1r+^ neurons, we subjected the differentially enriched genes of each subpopulation to a Gene Ontology (GO)-term analysis (Extended Data Fig. 9e–g). This analysis revealed that, specifically, transcripts in the GO terms neuropeptide signaling, regulation of response to nutrient levels and dendrite cytoplasm differed between both types of neurons (Extended Data Fig. 9e–g). To further investigate which neuropeptides and neuropeptide receptors are differentially expressed by POMC^Lepr+^ and POMC^Glp1r+^ neurons, the relative expression levels for all genes listed in the GO term ‘neuropeptide-signaling pathway’ (GO:0007218) were analyzed. While opioid receptors (*Oprd1* and *Oprm1*) and enrichment of neuropeptide Y (*Npy*) was higher in POMC^Glp1r+^ neurons, enrichment for prodynorphin (*Pdyn*), Cocaine and amphetamine regulated transcript protein (*Cartpt*), Neuromedin-U receptor 2 (*Nmur2*), and NPY-receptor (*Npy1r*) was higher in POMC^Lepr+^ neurons (Fig. 5d).

Next, we aimed to investigate whether and to which extent the genes enriched in either subpopulation identified by targeted ribosomal profiling might define clusters of POMC neurons as revealed by clustering in single-cell RNA-seq. The first data source was droplet single-cell RNA-seq of 20,921 cells from the ARC/median eminence^11^. Here, we filtered cells expressing *Pomc* (4,248/20,921 cells) and clustered them using the R Seurat package^24^. This yielded the identification of 11 clusters of *Pomc*-expressing neurons (Fig. 5e and Extended Data Fig. 9h). The second data source was a single-cell mRNA-seq dataset of 163 genetically marked *Pomc*-expressing neurons of mice yielding four clusters of POMC neurons^12^ (Fig. 5e). In Fig. 5e, we filtered the volcano plot of Fig. 5b for the markers of each cluster from the aforementioned datasets. For each of these clusters, we determined the log ratio of the marker genes differentially expressed in our ribosomal profiling between POMC^Lepr+^ and POMC^Glp1r+^ neurons (log ratio = log_2_ (*n*_Glp1r/*n*_Lepr)). We considered a cluster to be enriched for POMC^Glp1r+^ marker genes with a log ratio ≥ 2 and vice versa enriched for POMC^Lepr+^ marker genes with a log ratio ≤ −2. Applying this threshold to our clustering of POMC-positive neurons from the ARC/median eminence dataset identified clusters 2 and 10 as enriched for POMC^Lepr+^ marker genes and cluster 3 as enriched for POMC^Glp1r+^ marker genes (Fig. 5e). Similarly, we defined clusters 3 and 4 of the dataset of fluorescence-activated cell sorting (FACS)-purified POMC neurons enriched for POMC^Lepr+^ and clusters 1 and 2 enriched for POMC^Glp1r+^ marker genes (Fig. 5e). Collectively, our translational profiles of POMC^Lepr+^ and POMC^Glp1r+^ neurons allowed for successful independent identification of molecularly defined POMC clusters, which had been defined based on single-cell RNA-seq.

### Verification of endogenously expressed, differentially regulated genes in POMC^Lepr+^ and POMC^Glp1r+^ neurons

**Fig. 6:**
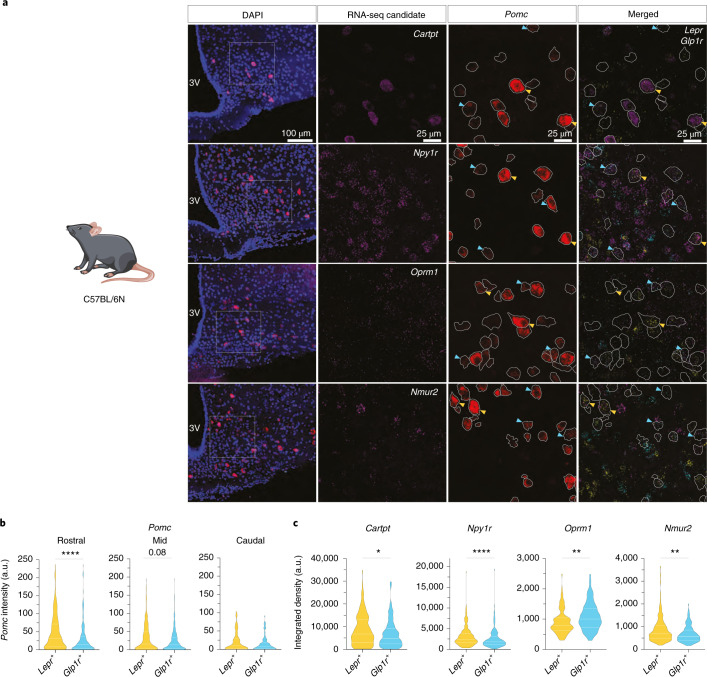
Differential expression of endogenous mRNAs for identified candidates in POMC^Lepr+^ and POMC^Glp1r+^ neurons. **a**, Representative microscopic images of RNA ISH against *Pomc*, *Glp1r* and *Lepr* together with differentially expressed neuropeptidergic signaling candidate RNAs identified in the ribotag experiments, *Cartpt*, *Npy1r*, *Oprm1* and *Nmur2*, in C57BL/6N mice at 12 weeks of age. First image shows ISH in the ARC with nuclear counterstain (blue, DAPI). Magnifications of the boxes (right) are shown with the indicated stainings. *Pomc*-positive neurons are outlined in white. Yellow and cyan arrows indicate *Lepr*-positive or *Glp1r*-positive POMC neurons, respectively. Scale bars represent 100 μm in the merged image and 25 μm in the magnifications. **b**, Violin plots showing the quantified intensity of *Pomc* mRNA across the rostrocaudal axis of the ARC in POMC^Lepr+^ or POMC^Glp1r+^ neurons as assessed from RNA ISH (Fig. 2a). Data are from *n* = 4 mice, with a minimum of four sections analyzed per animal. *Pomc*_Rostral_, *Lepr*^*+*^: Q_1_: 7.83, Q_2_: 31.12, Q_3_: 71.31; *Glp1r*^*+*^: Q_1_: 3.92, Q_2_: 14.86, Q_3_: 37.05, unpaired Mann–Whitney *U*-test, *Lepr*^+^ versus *Glp1r*^+^, *U* = 21,051, *P*_*uT*_ < 0.0001; *Pomc*_Mid_, *Lepr*^*+*^: Q_1_: 3.02, Q_2_: 15.71, Q_3_: 49.22; *Glp1r*^*+*^, Q_1_: 3.22, Q_2_: 13.43, Q_3_: 34.06, unpaired Mann–Whitney *U*-test, *Lepr*^+^ versus *Glp1r*^+^, *U* = 53,910, *P*_*uT*_ = 0.0796; *Pomc*_Caudal_, *Lepr*^*+*^: Q_1_: 9.46, Q_2_: 28.3, Q_3_: 103.5; *Glp1r*^*+*^ Q_1_: 8.03, Q_2_: 17.81, Q_3_: 91.96, unpaired Mann–Whitney *U*-test, *Lepr*^+^ versus *Glp1r*^+^, *U* = 3,467, *P*_*uT*_ = 0.06349. a.u., arbitrary units. **c**, Violin plots showing quantified expression of the RNA-seq candidates measured as integrated density in POMC^Lepr+^ or POMC^Glp1r+^ neurons as assessed from RNA ISH (**a**). *Cartpt*: *Lepr*^*+*^, Q_1_: 2,977, Q_2_: 6,729, Q_3_: 13,244, *Glp1r*^*+*^, Q_1_: 2,405, Q_2_: 4,741, Q_3_: 9,198, *Lepr*^+^ versus *Glp1r*^+^, *U*_MW_ = 9,252, *P*_*uT*_ = 0.0182; *Npy1r*: *Lepr*^*+*^, Q_1_: 1,609, Q_2_: 2,470, Q_3_: 3,887; *Glp1r*^*+*^, Q_1_: 1,154, Q_2_: 1,802, Q_3_: 3,069, *Lepr*^+^ versus *Glp1r*^+^, *U* = 22,352, *P*_*uT*_ < 0.0001; *Oprm1:* *Lepr*^*+*^, Q1: 631.6, Q2: 800.8, Q3: 1,046; *Glp1r*^*+*^, Q_1_: 729.2, Q_2_: 1,002, Q_3_: 1,360, *Lepr*^+^ versus *Glp1r*^+^, *U*_MW_ = 5017, *P*_*uT*_ = 0.001; *Nmur2*: *Lepr*^*+*^, Q_1_: 475.4, Q_2_: 689.7, Q_3_: 1,057; *Glp1r*^*+*^ Q_1_: 407.3, Q_2_: 574.9, Q_3_: 852, *Lepr*^+^ versus *Glp1r*^+^, *U*_MW_ = 8,760, *P*_*uT*_ = 0.0029. In **b** and **c**, solid white lines represent the median (Q_2_) and dashed white lines represent lower and upper quartiles (Q_1_ and Q_2_, respectively). *P* values were calculated using the unpaired Mann–Whitney (MW) *U*-test. *P ≤ 0.05, **P ≤ 0.01, ***P ≤ 0.001, ***P ≤ 0.0001.

We then aimed to validate the mRNA expression profiles of genes characterized as differentially enriched in genetically marked POMC^Lepr+^ and POMC^Glp1r+^ neurons via RNA ISH upon co-detection of endogenously expressed *Lepr* and *Glp1r* mRNA in wild-type mice (Fig. 6a). These analyses confirmed increased expression of *Pomc* in POMC^Lepr+^ neurons compared to POMC^Glp1r+^ neurons (Fig. 6b). Similarly, *Cartpt* expression was increased in POMC^Lepr+^ neurons compared to POMC^Glp1r+^ neurons (Fig. 6c). When we investigated the expression of exemplary genes encoding receptors for neuropeptides and energy-sensing signals, we confirmed the differential enrichment of *Npy1r*, *Oprm1* and *Nmur2* in POMC^Lepr+^ and POMC^Glp1r+^ neurons (Fig. 6c), as previously indicated by our RNA-seq results (Fig. 5d).

### POMC^Lepr+^ and POMC^Glp1r+^ neurons have distinct intrinsic electrophysiological properties

**Fig. 7:**
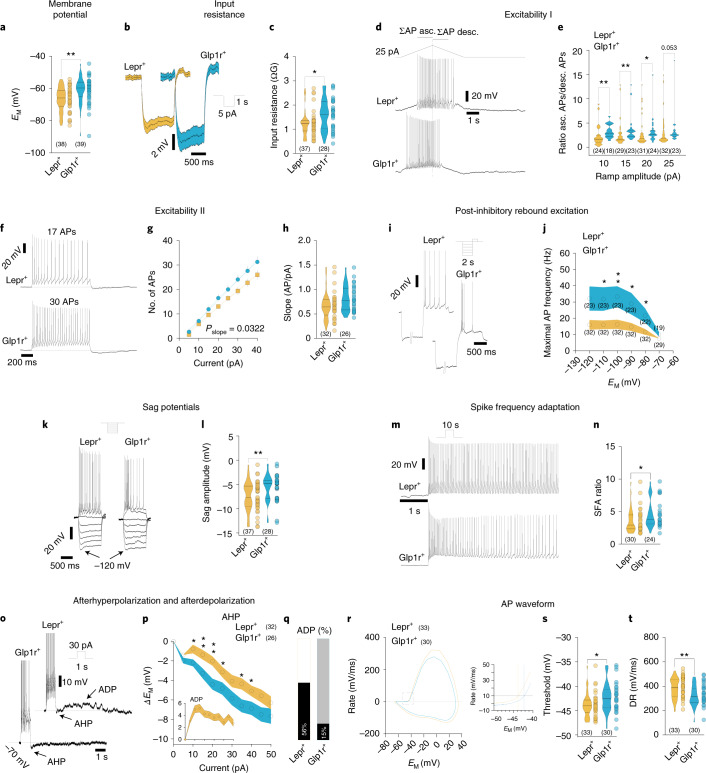
POMC^Lepr+^ and POMC^Glp1r+^ neurons have distinct intrinsic electrophysiological properties. **a**, Membrane potential (*E*_M_ ) of POMC^Lepr+^ and POMC^Glp1r+^ neurons (POMC^Lepr+^, *n* = 38, *E*_M_ = −66.72 ± 0.20 mV; POMC^Glp1r+^, *n* = 39, *E*_M_ = −61.20 ± 1.29 mV; *P*_MW_ = 0.001, *U* = 418). **b**,**c**, Input resistance. **b**, Mean response to 5-pA hyperpolarizing current pulses (POMC^Lepr+^, *n* = 37; POMC^Glp1r+^, *n* = 28) and violin plots (**c**) showing the input resistance of POMC^Lepr+^ and POMC^Glp1r+^ neurons (POMC^Lepr+^, *n* = 37, *R*_i_ = −1.24 ± 0.09 GΩ; POMC^Glp1r+^, *n* = 28, *R*_i_ = −1.61 ± 0.12GΩ; *P*_MW_ = 0.022, *U* = 345). **d**,**e**, Excitability I. Original recording illustrating a depolarizing ascending (asc.) and descending (desc.) current ramp protocol in POMC^Lepr+^ and POMC^Glp1r+^ neurons (**d**) and respective spike-number ratios of the ascending and descending phase of the protocol in POMC^Lepr+^ and POMC^Glp1r+^ neurons (**e**; 10 pA: POMC^Lepr+^, *n* = 24, r = 2.33 ± 0.45; POMC^Glp1r+^, *n* = 18, r = 3.35 ± 0.33; *P*_MW_ = 0.0019, *U* = 96.5, 15 pA: POMC^Lepr+^, *n* = 29, r = 2.81 ± 0.54; POMC^Glp1r+^, *n* = 23, r = 3.64 ± 0.58; *P*_MW_ = 0.0033, *U* = 176.5; 20 pA: POMC^Lepr+^, *n* = 31, *r* = 3.06 ± 0.55; POMC^Glp1r+^, *n* = 24, *r* = 3.34 ± 0.56; *P*_MW_ = 0.024, *U* = 239.5; 25 pA: POMC^Lepr+^, *n* = 32, *r* = 3.28 ± 0.59; POMC^Glp1r+^, *n* = 23, *r* = 3.41 ± 0.68; *P*_MW_ = 0.0527, *U* = 245.5). **f**–**h**, Excitability II. Example responses to 30-pA pulses (**f**). Number of action potentials (APs) as a function of current pulse amplitude (pA; **g**; POMC^Lepr+^, *n* = 32, slope = 0.687 ± 0.073 (AP/pA); POMC^Glp1r+^, *n* = 26, slope = 0.817 ± 0.096 (AP/pA); *P*_F_ = 0.0322, *F* = 4.62) in POMC^Lepr+^ and POMC^Glp1r+^ neurons and the respective slopes (**h**). **i**,**j**, Post-inhibitory rebound excitation. Original recordings illustrating the responses to a depolarizing current pulse that followed a prolonged (2 s) hyperpolarizing pre-pulse (**i**). Mean maximal instantaneous frequency as a function of the pre-pulse potential for POMC^Lepr+^ and POMC^Glp1r+^ neurons (**j**; −120mV: POMC^Lepr+^, *n* = 32, *f*_max_ = 14.93 ± 2.97 Hz; POMC^Glp1r+^, *n* = 23, *f*_max_ = 29.12 ± 7.14 Hz; *P*_MW_ = 0.0773, *U* = 264. −110 mV: POMC^Lepr+^, *n* = 32, *f*_max_ = 15.04 ± 2.70 Hz; POMC^Glp1r+^, *n* = 23, *f*_max_ = 29.83 ± 7.16 Hz; *P*_MW_ = 0.0374, *U* = 246. −100 mV: POMC^Lepr+^, *n* = 32, *f*_max_ = 15.29 ± 2.90 Hz; POMC^Glp1r+^, *n* = 23, *f*_max_ = 31.10 ± 6.38 Hz; *P*_MW_ = 0.0044, *U* = 203. −90 mV: POMC^Lepr+^, *n* = 32, *f*_max_ = 14.05 ± 2.53 Hz; POMC^Glp1r+^, *n* = 23, *f*_max_ = 29.75 ± 6.91 Hz; *P*_MW_ = 0.0374, *U* = 246. −80 mV: POMC^Lepr+^, *n* = 32, *f*_max_ = 11.48 ± 1.78 Hz; POMC^Glp1r+^, *n* = 22, *f*_max_ = 22.14 ± 4.54 Hz; *P*_MW_ = 0.017, *U* = 217. −70 mV: POMC^Lepr+^, *n* = 29, *f*_max_ = 7.46 ± 0.54 Hz; POMC^Glp1r+^, *n* = 19, *f*_max_ = 10.25 ± 1.46 Hz; *P*_MW _= 0.2081, *U* = 215). **k**,**l**, Sag potentials during hyperpolarization. Original recordings illustrating the response to five consecutive hyperpolarizing current pulses adjusted to reach −120 mV (**k**). Violin plots illustrating the sag amplitudes at hyperpolarization to −120mV (**l**; POMC^Lepr+^, *n* = 37, *E*_M_ = −7.46 ± 0.50 mV; POMC^Glp1r+^, *n* = 28, *E*_M_ = −5.69 ± 0.55 mV; *P*_MW_ = 0.0057, *U* = 311). **m**,**n**, SFA. **m**, Original traces illustrating the first 5 s of a response to a 10-s depolarizing current pulse in POMC^Lepr+^ and POMC^Glp1r+^ neurons. Violin plot showing SFA ratios (**n**) of POMC^Lepr+^ and POMC^Glp1r+^ neurons (POMC^Lepr+^, *n* = 30, *r* = 3.6 ± 0.35; POMC^Glp1r+^, *n* = 24, *r* = 4.66 ± 0.44; *P*_MW_ = 0.0233, *U* = 230). **o**–**q**, Afterhyperpolarization (AHP) and afterdepolarization (ADP). **o**, Original traces illustrating the slow AHP after 1-s depolarizing stimuli in POMC^Lepr+^ and POMC^Glp1r+^ neurons, and the ADP in POMC^Lepr+^ neurons. **p**, AHP amplitude for POMC^Lepr+^ and POMC^Glp1r+^ neurons as a function of the stimulus amplitude (5 pA, POMC^Lepr+^, *n* = 32, Δ*E*_M_ = −1.36 ± 0.19 mV; POMC^Glp1r+^, *n* = 26, Δ*E*_M_ = −1.97 ± 0.30 mV; *P*_*uT*_ = 0.084, *t* = 1.76, df = 56. 10 pA: POMC^Lepr+^, *n* = 32, Δ*E*_M_ = −0.77 ± 0.44 mV; POMC^Glp1r+^, *n* = 26, Δ*E*_M_ = −2.20 ± 0.36 mV; *P*_MW_ = 0.0156, *U* = 262. 15 pA: POMC^Lepr+^, *n* = 32, Δ*E*_M_ = −1.36 ± 0.50 mV; POMC^Glp1r+^, *n* = 26, Δ*E*_M_ = −3.28 ± 0.51 mV; *P*_*uT*_ = 0.0098, *t* = 2.675, df = 56. 20 pA: POMC^Lepr+^, *n* = 32, Δ*E*_M_ = −2.01 ± 0.48 mV; POMC^Glp1r+^, *n* = 26, Δ*E*_M_ = −4.38 ± 0.57 mV; *P*_MW_= 0.0044, *U* = 236. 25 pA: POMC^Lepr+^, *n* = 32, Δ*E*_M_ = −3.13 ± 0.53 mV; POMC^Glp1r+^, *n* = 26, Δ*E*_M_ = −4.82 ± 0.63 mV; *P*_*uT*_ = 0.0438, *t* = 2.06, df = 56. 30 pA: POMC^Lepr+^, *n* = 32, Δ*E*_M_ = −4.06 ± 0.63 mV; POMC^Glp1r+^, *n* = 26, Δ*E*_M_ = −5.82 ± 0.72 mV; *P*_*uT*_ = 0.0688, *t* = 1.86, df = 56. 35 pA: POMC^Lepr+^, *n* = 32, Δ*E*_M_ = −4.34 ± 0.59 mV; POMC^Glp1r+^, *n* = 26, Δ*E*_M_ = −6.60 ± 0.66 mV; *P*_*uT*_ = 0.0131, *t* = 2.56, df = 56. 40 pA: POMC^Lepr+^, *n* = 32, Δ*E*_M_ = −5.07 ± 0.55 mV; POMC^Glp1r+^, *n* = 26, Δ*E*_M_ = −6.86 ± 0.68 mV; *P*_*uT*_ = 0.043, *t* = 2.07, df = 56. 45 pA: POMC^Lepr+^, *n* = 32, Δ*E*_M_ = −5.58 ± 0.67 mV; POMC^Glp1r+^, *n* = 26, Δ*E*_M_ = −7.49 ± 0.73 mV; *P*_MW_ = 0.0659, *U* = 298. 50 pA: POMC^Lepr+^, *n* = 32, Δ*E*_M_ = −6.29 ± 0.67 mV; POMC^Glp1r+^, *n* = 25, Δ*E*_M_ = −7.66 ± 0.78 mV; *P*_MW_ = 0.3579, *U* = 342). Inset shows amplitude of the ADP, which was predominantly observed in POMC^Lepr+^ neurons. **q**, Percentage of POMC^Lepr+^ and POMC^Glp1r+^ neurons revealing ADPs after the 1-s excitatory stimuli (POMC^Lepr+^ *n* = 32; POMC^Glp1r+^ *n* = 26). **r**–**t**, Action potential waveform of POMC^Lepr+^ and POMC^Glp1r+^ neurons. Mean action potential phase plots; the region of the dashed rectangle is shown in higher resolution on the right (**r**). Action potential threshold, defined as when the rate in change of *E*_M_ reaches 10 mV/ms (**s**; POMC^Lepr+^, *n* = 33, *E*_M_ = −43.82 ± 0.43 mV; POMC^Glp1r+^, *n* = 30, *E*_M_ = −42.28 ± 0.55 mV; *P*_MW_ = 0.0212, *U* = 328) and depolarization rate (**t**; POMC^Lepr+^, *n* = 33, DR = −380.6 ± 14.5 mV/ms; POMC^Glp1r+^, *n* = 30, DR = −320.5 ± 15.6 mV/ms; *P*_*uT*_ = 0.0064, *t* = 2.83, df = 61). In all recordings, synaptic input was pharmacologically blocked (Methods). Error bars show ± s.e.m. ***P < 0.001; **P < 0.01; *P < 0.05. Bold lines in violin plots mark the median and light lines represent quartiles. *n*, number of cells recorded. DR, depolarization rate; *r*, ratio.

**Extended Data Fig. 10:**
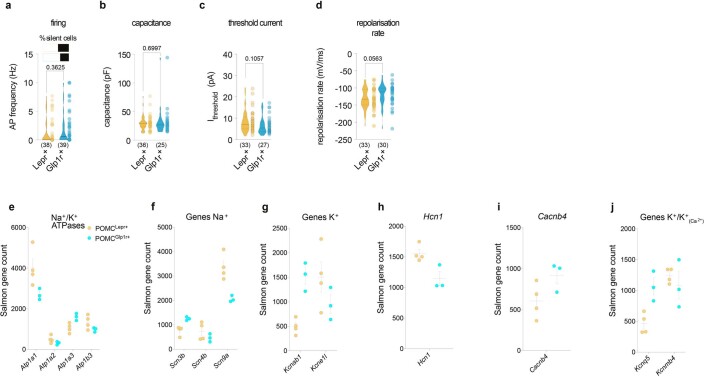
Spontaneous firing frequency, cell capacitance and significantly differentially enriched genes encoding ion channels and sub-units potentially contributing to different electrophysiological profiles of POMC^Lepr+^ and POMC^Glp1r+^ neurons. Violin plots illustrating spontaneous firing frequencies (**a**), whole-cell capacitance (**b**), threshold currents for the generation of action potentials (**c**), and the repolarisation rate of action potentials (**d**) of POMC^Lepr+^ and POMC^Glp1r+^ neurons. **e**, Genes encoding sodium-potassium-ATPases. **f**, Genes encoding voltage-gated Na^+^ channels and subunits modulating voltage-gated Na^+^ channels. **g**, Genes encoding voltage-gated K^+^ channels and subunits modulating voltage-gated K^+^ channels. **h**, Gene *Hcn1* hyperpolarization-activated cyclic-nucleotide-gated cation channels 1. **i**, Gene *Cacnb4* encoding a subunit modulating L-Type voltage-gated Ca^2+^ channels. **j**, Gene *Kcnq5* and *Kcnmb4* encoding a M-current K^+^ channel and the beta-subunit 4 modulating large conductance Ca^2+^ activated K^+^ channels, respectively. Data are represented as mean ± SEM. (**a**-**d**) *p*-values were calculated using the unpaired Mann-Whitney-U test. **e-j**, Selected from differentially ribotag-enriched transcripts analyzed in Fig. 5b (*p* ≤ 0.05). For POMC^Dre^ Lepr^Cre^ ROSA26lSlrSrEGFPL10a n = 4 hypothalami pooled from N = 24 mice, for POMC^Dre^ Glp1r^Cre^ ROSA26lSlrSrEGFPL10a n = 3 hypothalami pooled from N = 36 mice. *p*-values were calculated by DESeq2 1.26.0. Statistical details are given in Source data extended Fig. 7 Source data extended Fig. 7.

Next, we performed perforated patch-clamp recordings in genetically marked Lepr- and Glp1r-expressing POMC neurons in POMC^Dre^ Lepr^Cre^ ROSA26lSlrSrZsGreen^+/−^ or POMC^Dre^ Glp1r^Cre^ ROSA26lSlrSrZsGreen^+/−^ mice. Both neuronal populations have distinct functional properties and input–output relationships. While there were no differences in certain general properties including whole-cell capacitance, spontaneous firing rate and in the proportion of spontaneously active or silent neurons (Extended Data Fig. 10a,b), we found differences in key intrinsic electrophysiological properties (Fig. 7a–t). POMC^Glp1r+^ neurons were more depolarized (Fig. 7a), had a higher input resistance (Fig. 7b,c) and exhibited a higher excitability (Fig. 7d–h). During prolonged depolarization, they responded with pronounced phasic activity, while the POMC^Lepr+^ neurons were more tonically active (Fig. 7f,m,n).

During ascending and subsequently descending current ramps (Fig. 7d,e), POMC^Glp1r+^ neurons tended to have a lower threshold current (Extended Data Fig. 10c) and generated more action potentials during ascending ramps than the POMC^Lepr+^ neurons. During descending ramps, we observed the opposite; POMC^Lepr+^ neurons generated more action potentials than POMC^Glp1r+^ neurons. This resulted in a higher spike-number ratio between the ascending and descending ramps in POMC^Glp1r+^ neurons compared to POMC^Lepr+^ neurons, which indicates a voltage-dependent adaptation (Fig. 7e). These data suggest a phasic, adaptive excitability of POMC^Glp1r+^ neurons in response to sustained excitatory input, which cannot be explained simply by the differences in input resistance (Fig. 7b,c).

On a finer scale, we measured frequency–current relationships, post-inhibitory rebound, spike-frequency adaptation (SFA), and sag potentials during hyperpolarization from POMC^Glp1r+^ and POMC^Lepr+^ neurons, and found higher excitability in POMC^Glp1r+^ neurons, which was reflected by a ‘steeper’ frequency–current relationship (Fig. 7f–h).

We also observed a stronger post-inhibitory rebound excitation in POMC^Glp1r+^ (Fig. 7i,j). It is remarkable that we found profound post-inhibitory rebound excitation in POMC^Glp1r+^ neurons, while they generated smaller sag potentials than POMC^Lepr+^ neurons (Fig. 7k, l).

During depolarizing current pulses, POMC^Glp1r+^ cells underwent stronger SFA after the initial excitation (Fig. 7m,n), as reflected in a significantly higher SFA ratio (Fig. 7n). The high SFA ratio of POMC^Glp1r+^ neurons was accompanied by a marked afterhyperpolarization that followed depolarizations, for example, after trains of action potentials (Fig. 7o,p). This is mechanistically plausible since both SFA and afterhyperpolarization can be mediated by Ca^2+^-dependent K^+^ currents, which have been found in POMC neurons^25,26^. In contrast, in POMC^Lepr+^ neurons, which have a lower SFA and a clear tonic activity component during depolarizations, a sustained train of action potentials was followed by an afterdepolarization outlasting the excitatory input (Fig. 7o–q). A spike waveform analysis (Fig. 7r–t) showed a lower action potential threshold (Fig. 7r,s), a faster depolarization rate (Fig. 7r,t) and a trend toward a faster repolarization rate (Extended Data Fig. 10d) in action potentials of POMC^Lepr+^ neurons.

Since most active membrane properties are mediated by sets of ionic conductances, that is, multiple ion channel types, it is challenging to attribute these complex physiological properties causally to translational profiles. Nevertheless, we have identified cell-type-specific differences in the expression of ion channels (or subunits) that are considered regulators of some physiological properties that differ between POMC^Lepr+^ and POMC^Glp1r+^ neurons (Extended Data Fig. 10e–j). The phasic, adaptive excitability and the accompanying greater rebound properties of the POMC^Glp1r+^ neurons are in line with their comparatively higher expression of a modulatory β-subunit (*Cacnb4*; Extended Data Fig. 10i), which can lower the activation threshold and increase the conductance of voltage-gated Ca^2+^ channels^27^. The stronger SFA and afterhyperpolarization of POMC^Glp1r+^ cells compared to POMC^Lepr+^ neurons are consistent with the relatively high expression of the regulatory β4-subunit (*Kcnmb4*; Extended Data Fig. 10j) in POMC^Lepr+^ neurons, which decreases the conductance of big-conductance Ca^2+^-activated potassium channels^28^. Additionally, M-currents expressed by the *KCNQ* gene family can contribute to SFA and are upregulated in POMC^Glp1r+^ cells (Extended Data Fig. 10j)^29^. In agreement with the relatively depolarized membrane potential of POMC^Glp1r+^ cells and their relatively small sag potentials during hyperpolarization, we found low expression of Na^+^/K^+^ ATPases (*Atp1a1*) and hyperpolarization-activated cyclic-nucleotide-gated cation channel 1 (*Hcn1*), respectively (Extended Data Fig. 10e, h)^30^.

In summary, we have revealed cell-type-specific electrophysiological differences between POMC^Lepr+^ and POMC^Glp1r+^ neurons, which are paralleled by the cell-type-specific expression of ion channels and receptors for energy homeostasis-related signals.

### Specific regulation of POMC^Lepr+^ and POMC^Glp1r+^ neurons by energy-state-sensing signals

**Fig. 8:**
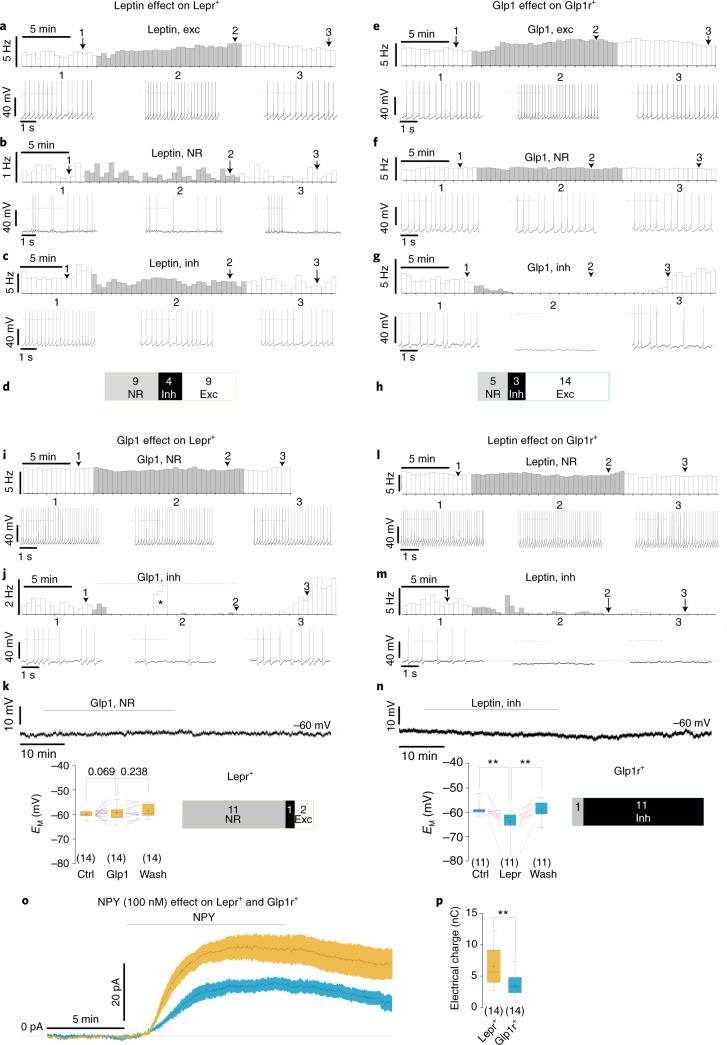
Leptin and Glp1 differentially modulate POMC^Lepr+^ and POMC^Glp1r+^ neurons and exhibit differences in NPY-induced currents. **a**–**h**, Effect of leptin on POMC^Lepr+^ neurons (**a**–**d**) and Glp1 on POMC^Glp1r+^ neurons (**e**–**h**). Rate histograms and respective original recordings illustrating the effect of leptin on POMC^Lepr+^ neurons (**a**–**c**) and the effect Glp1 on POMC^Glp1r+^ neurons (**e**–**g**). Each figure shows a single example each of a peptide-excited, a peptide-inhibited and a nonresponsive neuron. **d**,**h**, Numbers of peptide-responsive neurons in the respective cell types. **i**–**n**, Effect of Glp1 on POMC^Lepr+^ neurons (**i**–**k**) and leptin on POMC^Glp1r+^ neurons (**l**–**n**). **i**,**j**,**l**,**m**, Rate histograms and respective original recordings showing single examples of neurons that were nonresponsive or inhibited by the respective peptides. The asterisk in the rate histogram (**j**) reflects action potentials that were elicited by current protocols. **k**,**n**, Current-clamp recordings, in which action-potential-induced synaptic release is suppressed by TTX (1 µM). Top: original recordings. Bottom left: summary and quantification of all recordings. Red lines indicate recordings with significant changes in membrane potentials. The population responses were compared by using one-way ANOVA with Tukey’s post hoc test (**k**: POMC^Lepr+^, *n* = 14, control (ctrl) versus Glp1 *P* = 0.0692, Glp1 versus wash *P* = 0.238, ctrl versus wash *P* = 0.0218, *F* = 6.40; **n**: POMC^Glp1r+^, *n* = 12, ctrl versus leptin *P* = 0.0067, leptin versus wash *P* = 0.008, ctrl versus wash *P* = 0.722; *F* = 8.72; **P < 0.01). Box plots were generated according to Tukey’s test, where ‘+’ illustrates the mean. Bottom right: numbers of peptide-responsive neurons in the respective cell types. **o**,**p**, Effect of NPY on POMC^Lepr+^ neurons and POMC^Glp1r+^ neurons. **o**, Voltage-clamp recordings. NPY induced inward currents in POMC^Lepr+^ neurons (*n* = 14; yellow) and POMC^Glp1r+^ neurons (*n* = 14; blue), shown as the mean ± s.e.m. **p**, Electrical charge that flowed during 10 min of NPY application (POMC^Lepr+^, *n* = 14, minima = 2.73, Q_1_ = 4.03, Q_2_ = 5.68, Q_3_ = 9.19, maxima = 12.24, mean ± s.e.m. = 6.54 ± 0.80 nC; POMC^Glp1r+^, *n* = 14, minima = 0.78, Q_1_ = 2.34, Q_2_ = 3.274, Q_3_ = 4.82, maxima = 7.32, mean ± s.e.m. = 3.53 ± 0.48 nC; *P*_*uT*_ = 0.0034, *t* = 3.23, df = 26, two-tailed unpaired Student’s *t*-test. **P < 0.01). In the box plots, a ‘+’ sign show the mean and the horizontal line is the median. The whiskers were calculated according to the Tukey method. In all recordings, synaptic input was pharmacologically blocked (Methods). Peptides were bath applied at the indicated concentrations: leptin (100 nM), Glp1 (300 nM) and NPY (100 nM). Responsiveness of individual neurons was defined by the 3-σ criterion (Methods). *n*, number of cells recorded. exc, excited; inh, inhibited; NR, not responsive. Q_1_, Q_2_ and Q_3_ represent the lower quartile, median and upper quartile, respectively.

Most neurons showed significant but mostly subtle modulatory effects to stimulation with the cognate ligands of their defining receptors, that is, leptin (Fig. 8a–d) and Glp1 (Fig. 8e–h). The modulatory effects were often differential and not homogeneous within one cell type, and we also observed modulation upon stimulation with the non-cognate ligands (Fig. 8i–n).

Leptin (100 nM) excited 40% and inhibited 20% of POMC^Lepr+^ neurons, while the remaining 40% of these cells were not responsive (Fig. 8a–d). In 60% of POMC^Glp1r+^ neurons, leptin did not affect the neuronal activity, while, despite the absence of Lepr expression, it inhibited 40% of these cells (Fig. 8l,m). Thus, in the presence of GABA receptor and glutamate receptor blockers, leptin modulated both neuronal populations differentially.

Correspondingly, Glp1 (300 nM) excited most (63%) POMC^Glp1r+^ neurons, whereas only 13% of the neurons were inhibited, and 22% were not affected by Glp1 (Fig. 8e–h). POMC^Lepr+^ neurons, which largely do not express the Glp1r, were mostly unaffected by Glp1 (81%), and only 18% of these neurons were inhibited (Fig. 8i, j).

While the distinctive responses of both cell types to the two ligands indicate that classification of these neurons according to Lepr and Glp1r expression defines different responses to two satiety-communicating signals, the relatively high variability of the effects also raises the question of whether interactions within the energy homeostasis-regulating network contribute to the modulatory effects. Consequently, we performed recordings where, in addition to blocking GABA and glutamate receptors, action-potential-dependent signaling was suppressed by tetrodotoxin (TTX; 1 µM). Around 80% of POMC^Lepr+^ neurons remained unaffected by Glp1, while 14% of neurons depolarized and 6% hyperpolarized (Fig. 8k). The proportion of POMC^Lepr+^ neurons that responded to Glp1 is consistent with the proportion of these cell types expressing both Lepr and Glp1r, although lower than that previously reported to respond to Glp1, where 37% of alternatively identified POMC^Lepr+^ neurons responded to a higher Glp1 dose (1 µM)^31^. Approximately 91% of POMC^Glp1r+^ neurons responded with a reversible hyperpolarization, while only 9% of neurons were unaffected by leptin (Fig. 8n). At first sight, these data might imply that leptin has direct effects on POMC^Glp1r+^ neurons (and Glp1 directly affects POMC^Lepr+^). Alternatively, the results could indicate modulation of spontaneous release^32^. Moreover, these experiments indicate that the modulation of POMC neurons might lead to stronger cross-interactions between POMC^Lepr+^ and POMC^Glp1r+^ neurons than originally expected.

Furthermore, consistent with a higher level of the *Npy1r* in POMC^Lepr+^ neurons (Figs. 5d and 6c), NPY (100 nM) elicited larger outward currents in POMC^Lepr+^ cells compared to POMC^Glp1r+^ neurons (Fig. 8o,p). Collectively, differentially expressed neuropeptide receptors, as identified via translational profiling, accurately predicted differential pharmacological responses of these cell types.

## Discussion

Recent developments in single-cell RNA-seq have largely extended our view on the heterogeneity of molecularly defined neurocircuits, including those in control of energy and glucose homeostasis^11^. However, defining the underlying molecular heterogeneity of distinct neurocircuits, their functional organization and output remains a largely unmet territory in modern neuroscience. Here, we provide new mouse models, which allow for functional interrogation of heterogeneous populations based on combinatorial Cre- and Dre-dependent recombination in vivo. Recombinase-based gene targeting has become a valuable tool not only in neuroscience allowing for the modulation of specific cell types mostly via Cre-loxP-mediated recombination^13^. Alternative recombinase systems such as FLP/FRT-mediated recombination provide complementary tools, but are limited through a lower efficiency of recombination in mice despite that improved versions have been developed (FLPe and FLPo)^33^. While FLPe has been used for successful combinatorial recombination-based targeting of heterogeneous dopamine neurons using viral approaches, only a few examples have used FLP-based recombination for intersectional targeting in transgenic mice^34^.

An alternative recombinase system has been identified, that is, Dre-rox-mediated recombination^17^. Previous studies had indicated the feasibility of combinatorial Cre/Dre-recombinase usage for targeting specific neuronal subpopulations^16^. Here, we have systematically expanded the repertoire of tools and validated mice, enabling use of this system to define specific heterogeneous cell types. First, we show that Dre-based recombination allows for efficient targeting of rox-marked transgenes in single-allele configuration. Moreover, we show that Dre-dependent recombination is specific for rox-flanked DNA segments, as we have observed no cross-reactivity with loxP-flanked alleles in several lines of transgenic mice. While it had previously been reported that Dre-mediated recombination may result in background recombination of loxP-flanked DNA in a ROSA26lSlTomato reporter strain^16^, our extensive experiments clearly validate the usage of Cre/Dre-mediated combinatorial recombinases for complementary analyses of heterogeneous populations in addition to FLP-based approaches.

Nevertheless, Dre-dependent recombination appears to be less efficient compared to Cre-dependent recombination. Of the numerous, independent POMC^Dre^ transgenic mouse lines employing the exact same POMC BAC construct to express Dre as previously used to express Cre in these cells, the efficiency of Dre-mediated recombination is lower than Cre-mediated recombination, since the maximum rate of recombination was lower and increased over a longer period of development^4^. However, this represents a crucial advantage, when it allows Dre-mediated recombination to bypass narrow developmental periods, where a promoter is temporarily active during development in a subset of cells, and which thus unfaithfully marks cells upon very efficient recombinase targeting. This has been exemplified for POMC cells, where efficient POMC^Cre^-mediated recombination during development targets cells, which later during development acquire a functionally antagonistic phenotype, that is, one of AgRP neurons^18^. In contrast, the lower efficiency and prolonged onset of POMC^Dre^-mediated recombination enables circumvention of this effect and thus allows for long-term genetic marking of *bona fide* POMC-expressing cells.

We have successfully used combinatorial Cre/Dre-dependent recombination to genetically mark heterogeneous POMC neuron populations for fluorophore expression and translational profiling. Expression of ZsGreen in the nucleus of targeted cells allowed for efficient labeling of cell bodies and subsequent anatomical reconstruction of distribution probabilities of distinct cell types within the limited space of the ARC upon tissue clearing^22^. Here we have developed an image analysis pipeline that enables high-precision and high-resolution mapping of cell localization onto a standardized distribution space. Thereby, we have defined distribution maps of heterogeneous POMC cell populations within the ARC, revealing that POMC^Lepr+^ and POMC^Glp1r+^ cells clearly exhibit a distinct anatomical distribution.

In addition, our experiments provide numerous new insights into the functional organization of hypothalamic POMC neurocircuits. First, the role of leptin in the regulation of POMC neurons has been subject to controversial findings over the last few years. Deletion of leptin receptors from POMC neurons throughout development causes mild obesity and hyperphagia, while the same intervention during adulthood has no effect on body weight or energy homeostasis^4,35^. On the other hand, POMC deficiency or selective ablation of hypothalamic POMC neurons in mice^36^ and POMC deficiency in humans causes massive obesity^37^, and re-expression of POMC in Lepr-expressing cells is sufficient to restore this effect in mice^38^, providing evidence for a role of POMC^Lepr+^ neurons in the control of energy homeostasis. However, selective chemogenetic stimulation of POMC^Lepr+^ neurons in our study only induces a minor suppression of food intake. Collectively, these data support a predominantly developmental role of Lepr-expressing POMC neurons in the control of energy homeostasis, but at the same time indicate that additional regulators other than the direct action of leptin contribute to control their activity. Recent experiments using Ca^2+^ imaging of POMC neuron activity in mice, have revealed that POMC neurons rapidly change their activity in response to sensory food perception, independent of changes in circulating energy-state-sensing hormones, and that this regulation contributes to the priming of liver ER adaptation upon sensory food perception^39^. Thus, top-down control of POMC^Lepr+^ neurons or additional energy-state-sensing signals may also represent functionally relevant regulators of POMC^Lepr+^ neuron activity.

In contrast, chemogenetic activation of POMC^Glp1r+^ neurons induces a more dramatic food intake suppression than what has previously been described, when POMC^Cre^-expressing neurons have been activated either chemogenetically or optogenetically^40^. This is remarkable since transgenically targeted POMC^Glp1r+^ neurons represent a significantly smaller population than the POMC^Lepr+^ neurons (data not shown). While POMC neurons were previously reported to be both GABAergic or glutamatergic, our gene expression and in situ analyses indicate low levels of *Slc17a6* (*Vglut2*) expression in both neuronal groups.

Surprisingly, the chemogenetic activation of POMC^Lepr+^ and POMC^Glp1r+^ neurons in female mice had no effect on food intake. ISH analysis showed that these neurons were also efficiently activated in females (Extended Data Fig. 5j). Thus, it is likely that the regulation of food intake in males and females is controlled differently, which is corroborated by previous reports of higher expression of *Pomc* mRNA and higher firing rates of POMC neurons in female mice compared to males^41^.

Another possibility for why selective activation of POMC^Glp1r+^ neurons may have a more pronounced food intake-suppressing effect than that previously reported using a global POMC population stimulation is evidenced by our electrophysiological studies. Typically, we observed differential and nonhomogeneous modulatory effects within one neuronal type, even in the presence of glutamate receptor and GABA receptor blockers. These findings raise the question whether factors other than glutamatergic and GABAergic interactions within this regulatory network might contribute to the modulation. One reason why this is a crucial question is that we have observed modulatory effects of ligands whose associated receptors are not expressed in the respective cell types. POMC^Glp1r+^ neurons, for example, are inhibited during leptin application, even with glutamate and GABA receptor blockers present, and when action-potential-dependent synaptic release is suppressed (Fig. 8n). Since we mainly did not detect the corresponding receptors in the majority of these neurons, this could indicate that leptin induces the release of inhibiting mediators from presynaptic neurons in a non-action-potential-dependent manner. These mediators might be released by modulation of spontaneous release^32^. In primary spinal afferents, activation of TRPV1 channels facilitates asynchronous synaptic release presumably by presynaptic Ca^2+^ influx^42^. Thus, it is plausible to consider that leptin mediates activation of TRP channels as previously described^43^ and thereby induces or increases the spontaneous release of neuronal mediators via increased Ca^2+^ influx.

In line with the notion of alternative regulators of POMC^Lepr+^ and POMC^Glp1r+^ neuronal activity, our translational profiling has allowed the identification of numerous potentially differential regulators of these neuronal classes besides leptin and Glp1. Here, POMC^Lepr+^ neurons in particular express numerous receptors for additional energy-state-sensing signals, which have already been validated to suppress feeding and even reduce body weight in obesity. Interestingly, our study reveals the differential expression of *Npy1r* and *Npy5r* in distinct POMC neuron populations. Given that the primary source of NPY for POMC neurons in the ARC is neighboring AgRP neurons coexpressing NPY, these findings may point to the possibility that POMC^Lepr+^ neurons and POMC^Glp1r+^ neurons are under differential inhibitory control by AgRP neurons. Indeed, we have also electrophysiologically validated that NPY induces a larger (inhibitory) outward current in POMC^Lepr+^ neurons, compared to POMC^Glp1r+^ neurons (Fig. 8o,p). Moreover, the surprising identification of the expression of NPY in POMC^Glp1r+^ neurons also warrants further study.

In summary, we have employed new models to begin to shed light on the anatomical, electrophysiological, molecular and functional heterogeneity of critical metabolism-regulatory POMC neurons. The detailed insights may also aid the development of new, rationalized strategies for the therapeutic manipulation of the melanocortin circuitry.

## Methods

### Animals and animal care

All animal procedures were conducted in compliance with protocols approved by local government authorities (Bezirksregierung Köln). Permission for breeding and experiments on mice was issued by the Department for Environment and Consumer Protection-Veterinary Section in Cologne ((§11) 576.1.35.2.G 07/18, 84-02.04.2017.A058). Mice were housed in individually ventilated cages at 22 –24 °C using a 12-h light/dark cycle. Animals had access to water and food ad libitum. Unless otherwise stated, animals were fed a normal chow diet (NCD; Teklad Global Rodent 2018, Harlan) containing 53.5% carbohydrate, 18.5% protein and 5.5% fat (12% of calories from fat). The high-fat diet (HFD; C1057, Altromin) consisted of 32.7% carbohydrate, 20% protein and 35.5% fat (55.2% of calories from fat).

### Generation of genetically modified mouse strains

#### Driver lines

The Lepr^Cre^ and Glp1r^Cre^ mouse lines have been previously described^21,44^. Lepr^Cre^ mice and the Glp1r^Cre^ lines were kindly provided by M. G. Myers and F. Reimann, respectively.

The POMC^Dre^ BAC construct was generated by inserting the sequence encoding Dre recombinase together with a kanamycin/neomycin resistance cassette from the pTEDre plasmid into the start codon of the *POMC* gene of RP11-124K7 BAC via Red/ET recombination. Primers containing POMC-specific homology arms, 5POMC-Dre: 5′-tccctccaatcttgtttgcctctgcagagactaggcctgacacgtggaaggccaccatgggtaagaagaaga-3′ and 3POMC Dre: 5′-accagctccacacatctatggaggtctgaagcaggagggccagcaacagggaggatttaatatttctgacgc-3′, were utilized for amplification. The ATG codon from POMC was replaced by that of the Dre recombinase. The BAC was linearized with PISceI in the presence of 2,5 mM spermidine and loaded onto a self-assembled CL-4b sepharose column (Sigma, CL4B200), equilibrated with injection buffer (5 mM Tris, 0.1 mM EDTA (pH 7.6)). The flow-through fraction with the highest concentration of digested BAC was chosen for pro-nucleus injection, performed by the team of R. Naumann at the MPI for Molecular Cell Biology and Genetics in Dresden. Cre and Dre transgenic animals were bred to C57BL/6N mice for maintenance in the mouse facility of the Max Planck Institute for Metabolism Research in Germany.

#### ROSA26 transgenic mouse lines

Generation of ROSA26lSlrSrZsGreen (ROSA26-CAGS-lox-STOP-lox-rox-STOP-rox-ZsGreen) mice has been described in a previous study^20^. These mice have been crossed to a ubiquitously expressed Deleter-Cre line to obtain R26rSrZsGreen mice^20^. ROSA26rSrlSltdTomato (ROSA26-CAGS-rox-STOP-rox-lox-STOP-lox-tdTomato-WPRE) mice were purchased from The Jackson Laboratory.

The knock-in lines ROSA26lSlrSrZsGreen (ROSA26-CAGS-lox-STOP-lox-rox-STOP-rox-ZsGreen), ROSA26lSlrSrhM3Dq (ROSA26-CAGS-lox-STOP-lox-rox-STOP-rox-hM3Dq-2A-ZsGreen-WPRE) and ROSA26lSlrSrEGFPL10a (ROSA26-CAGS-lox-STOP-lox-rox-STOP-rox-EGFPL10a-WPRE) were generated in-house. For generation of these mouse lines, a ROSA26 locus-targeting vector (B9-36) was designed in which both a loxP-flanked STOP cassette and a rox-flanked STOP cassette prevent CAGS promoter-driven expression of the corresponding functional transgenic construct. For hM3Dq, the 5′-primer (5AscRassle) overhang used for the amplification of the transgene contained an AscI site and a Kozak consensus sequence and the 3′-primer (3AscRassle) overhang contained an AscI site and one C to stay in frame for the 2A-ZsGreen translation. For the EGFPL10a, the 5′-primer overhang used for the amplification of the transgene contained an AscI site and a Kozak consensus sequence and the 3′-primer overhang contained an XmaI. The sequence-verified knock-in sequences of hM3Dq and EGFPL10a were cloned into the AscI-digested and AscI/XmaI-digested B9-36-targeting constructs, respectively. After vector transfection into Bruce 4 embryonic stem cells, they were subsequently screened for correct integration by standard Southern blot methods. To this end, a ROSA26 probe was used^45^ on EcoRI-digested clonal genomic DNA that indicated homologous recombination via detection of an additional 7.1-kb band besides the 16-kb endogenous ROSA26 band. To exclude random integration, a Neo probe was used that detected one single 7.1-kb band in case of a single correct ROSA26 insertion. Correctly targeted and verified embryonic stem cell clones were chosen for blastocyst injection carried out by Taconic Biosciences to obtain chimeric animals. Resulting chimeras were backcrossed with C57BL/6N mice to obtain germline transmission on a pure C57BL/6N background.

#### Generation of experimental lines

For the POMC^Dre^ ROSA26rSrZsGreen mouse line, the breeding scheme consisted of mating heterozygous POMC^Dre^ mice to homozygous ROSA26^rx/rx^ mice of the ZsGreen construct. Resulting double transgenic Dre^+/−^ ROSA26^rx/wt^ mice were used as experimental animals and compared to C57BL/6N mice for metabolic phenotyping. Littermates of both sexes were used for experiments.

The mouse lines POMC^Dre^ Lepr^Cre^ ROSA26lSlrSrZsGreen, POMC^Dre^ Glp1r^Cre^ ROSA26lSlrSrZsGreen, POMC^Dre^ Lepr^Cre^ ROSA26rSrlSltdTomato, POMC^Dre^ Glp1r^Cre^ ROSA26rSrlSltdTomato, POMC^Dre^ Lepr^Cre^ ROSA26lSlrSrhM3Dq, POMC^Dre^ Glp1r^Cre^ ROSA26lSlrSrhM3Dq, POMC^Dre^ Lepr^Cre^ ROSA26lSlrSrEGFPL10a and POMC^Dre^ Glp1r^Cre^ ROSA26lSlrSrEGFPL10a were generated via mating heterozygous double transgenic mice to homozygous ROSA26^fl;rx/fl;rx^ mice of the corresponding functional transgene construct (Extended Data Fig. 2b). Resulting triple transgenic Cre^+/−^ Dre^+/−^ ROSA26^fl;rx/wt^ mice were used as experimental animals and compared to genotype controls as stated in the figure legends. Littermates of both sexes were used for experiments.

The C57BL/6N mouse line was purchased from Charles River. For metabolic phenotyping, both genders were used for experiments as indicated in the figure legends.

### Experimental details

#### Glucose tolerance test

Glucose tolerance tests were performed at 13 weeks of age with 16-h fasted mice. Body weights of mice and their basal blood glucose using a glucometer and glucose strips (Contour Next, Bayer HealthCare) were determined before the start of the experiment. Mice were injected with 20% glucose, and blood glucose was measured at 15, 30, 60 and 120 min after injection.

#### Nuclear magnetic resonance

Lean and fat mass were determined via nuclear magnetic resonance (NMR Analyzer Minispec mq 7.5, Bruker Optik) in live mice. Alternatively, body composition was analyzed by computed tomography (CT) in isoflurane-anesthetized mice (Dräger and Piramal Healthcare). For data acquisition on an IVIS Spectrum CT scanner (Caliper LifeScience), we used IVIS LivingImage Software V.4.3.1. Quantification of lean and fat mass contents were determined with a modification of the previously described VINCI software package 4.61.0.

#### Indirect calorimetry

Metabolic phenotyping and food intake were measured by an automated PhenoMaster open-circuit indirect, calorimetry system (TSE Systems). Mice were allowed to acclimatize to the experimental setup for 4 d before the start of each experiment. Food and water were available ad libitum. Data acquisition was carried out by TSE Phenomaster versions 6.2.5 and above.

### Study design of DREADD animals

POMC^Dre^ Lepr^Cre^ ROSA26lSlrSrhM3Dq and POMC^Dre^ Glp1r^Cre^ ROSA26lSlrSrhM3Dq mice and corresponding genotype controls were characterized at 13–15 weeks of age with ad libitum access to NCD. Saline (0.9%) or CNO injections (3 mg per kg body weight) were administered intraperitoneally. For measurements of food intake (as described under ‘Indirect calorimetry’), mice were injected with saline at 18:00 and 23:00, followed by a 1-d recovery period and subsequent CNO injections at 18:00 and 23:00 on the next day. For measurements of energy expenditure, respiratory exchange ratio and locomotion (as described under ‘Indirect calorimetry’), mice were injected with saline at 17:00, 22:00 and 07:00 followed by CNO at 17:00, 22:00 and 07:00 on the next day. Before perfusions at 22–26 weeks of age, mice were fasted for 2 h and injected with saline or CNO 1 h before perfusion. Serum for the corticosterone enzyme-linked immunosorbent assay (ELISA) was obtained from mice fasted for 2 h and injected with CNO 1 h before blood collection. Littermates of both sexes were used for experiments as indicated in text and figures.

### CNO administration

CNO (Abcam, ab141704) powder was dissolved in dimethyl sulfoxide (DMSO; 100 mg ml^−1^) and diluted at a ratio of 1:333 in 0.9% NaCl (saline).

### Study design of bacTRAP (EGFPL10a) mice

For POMC^Dre^ Lepr^Cre^ ROSA26lSlrSrEGFPL10a and POMC^Dre^ Glp1r^Cre^ ROSA26lSlrSrEGFPL10a animals, 6 triple transgenic bacTRAP mice (3 females and 3 males) and 12 triple transgenic bacTRAP mice (6 females and 6 males) were pooled for each replicate, respectively, accounting for three to four replicates per POMC subpopulation. Mice were killed at 12 weeks of age in a random-fed state by decapitation. Whole hypothalami were obtained using a mouse brain slicer matrix and snap frozen in liquid nitrogen until translating ribosome affinity purification (TRAP).

### Perfusion and tissue fixation

With the exception of DREADD animals, all mice were perfused in a random-fed state. Mice were deeply anesthetized and perfused transcardially with 1× PBS followed by ice-cold 4% paraformaldehyde (PFA; in 1×PBS; pH 7.4). The brain was removed from the skull and post-fixed in 4% PFA at 4 °C for approximately 24 h, and then moved to 20% sucrose solution (in 1× PBS) at 4 °C. The brains were cut at 20 μm on a sliding microtome (Leica Microsystems, SM2010R) equipped with a stage for dry ice. For immunohistochemistry, sections were either collected on slides or in bins containing anti-freeze solution (30% ethylene glycol and 20% glycerol in PBS), and subsequently stored at −20 °C until further processing. For RNA ISH, sections were mounted on SuperFrost Plus Gold slides (Thermo Fisher Scientific, 11976299) and subsequently stored at −80 °C until further processing.

### Immunohistochemistry

For immunofluorescence stainings against ZsGreen, POMC, tdTomato and EGFP, all incubation steps were performed at room temperature unless otherwise stated.

Floating sections were washed once for 10 min in PBS, incubated for 10 min in 0.3% glycine, washed again for 5 min in PBS and incubated for 10 min in 0.03% SDS/PBS. Subsequently, sections were blocked for 1 h in 3% donkey serum in PBS containing 0.25% Triton X. Next, sections were incubated overnight at 4 °C in primary antibody diluted in Signal Stain (Cell Signaling, 8114). Primary antibodies and dilutions used were: rabbit anti-ZsGreen (Takara Bio Clontech, 632474; 1:100), rabbit anti-POMC (Phoenix, H-029-30; 1:1,000), rat anti-mCherry (for tdTomato; Thermo Fisher Scientific, M11217; 1:1,000) and chicken anti-GFP (Abcam, ab13970; 1:1,000) were used. The following morning, sections were washed three times for 10 min in PBS containing 0.1% Triton X and incubated for 1 h in secondary antibody in PBS containing 0.25% TritonX. Secondary antibodies and dilutions were donkey anti-rabbit Alexa Fluor 488 (Thermo Fisher, A21206; 1:500), donkey anti-rat Alexa Fluor 594 (Jackson ImmunoResearch, 712-585-153; 1:500) or goat anti-rabbit Alexa Fluor 594 (Thermo Fisher Scientific, A11012; 1:500) and goat anti-chicken Alexa Fluor 488 (Thermo Fisher Scientific, A11039; 1:500), for 1 h at room temperature. After three washing steps for 10 min in PBS containing 0.1% Triton X, sections were mounted in Vectashield DAPI-containing mounting medium (Vector Laboratories, VEC-H-1200) and stored at 4 °C in the dark until imaging.

### Corticosterone ELISA

Concentrations were determined using a commercial Corticosterone ELISA kit from CrystalChem as described in the user’s manual (80556).

### Imaging and quantification of immunohistochemistry

Images were captured using a confocal Leica TCS SP-8-X microscope, equipped with a ×40/1.30 oil objective with the acquisition software (Leica ASX V.3.5.5.19976). Next, *z*-stacks were taken with optical sections of 0.9 μm. Laser intensities were kept constant throughout all related conditions. Images were imported into FIJI where maximum intensities were projected. For representative images, adjustments in brightness and contrast for each channel were kept constant throughout all related conditions, whereas for quantifications of POMC and tdTomato signals, all channels were kept unmodified and one to four sections were quantified per mouse and area. Images were converted to 8-bit, and the threshold for signal detection for each channel was determined by visual judgment and consistently applied to all images. ROIs were defined around corresponding anatomical locations and raw integrated densities measured within ROIs for POMC and tdTomato signals.

### RNA in situ hybridization

The fluorescence ISH technique (RNAscope) was used to detect mRNA of *Pomc*, *Agrp*, *Lepr-tv1*, *Glp1r*, *ZsGreen*, *Fos*, *Cartpt*, *Vglut2*, *Vgat*, *Npy1r*, *Oprm1* and *Nmur2*. All reagents were purchased from Advanced Cell Diagnostics (ACD) if not otherwise stated. The *Pomc* probe (314081) contained ten oligonucleotide pairs targeting region 19–995 (NM_008895.3) of the *Pomc* transcript; the *Agrp* probe (400711) contained 16 oligonucleotide pairs targeting region 11–764 (NM_001271806.1) of the *Agrp* transcript; the *Lepr-tv1* probe (471171) contained 19 oligonucleotide pairs targeting region 3220–4109 (NM_146146.2) of the *Lepr* transcript variant 1; the *Glp1r* probe (418851) contained 20 oligonucleotide pairs targeting region 108–1203 (NM_021332.2) of the *Glp1r* transcript; the *ZsGreen* probe (461251) contained 15 oligonucleotide pairs targeting region 980–1655 (JQ071441.1) of the *ZsGreen* transcript; the *Fos* probe (316921) contained 20 oligonucleotide pairs targeting region 407–1427 (NM_010234.2) of the *Fos* transcript; the *Cartpt* probe (432001) contained 17 oligonucleotide pairs targeting region 11–860 (NM_013732.7) of the *Cartpt* transcript; the *Vglut2* probe (319171) contained 20 oligonucleotide pairs targeting region 1986–2998 (NM_080853.3) of the *Vglut2* transcript; the *Vgat* probe (319191) contained 20 oligonucleotide pairs targeting region 894–2037 (NM_009508.2) of the *Vgat* transcript; the *Npy1r* probe (427021) contained 20 oligonucleotide pairs targeting region 227–1169 (NM_010934.4) of the *Npy1r* transcript; the *Oprm1* probe (315841) contained 20 oligonucleotide pairs targeting region 1135–2162 (NM_001039652.1) of the *Oprm1* transcript; and the *Nmur2* probe (314111) contained 20 oligonucleotide pairs targeting region 69–1085 (NM_153079.4) of the *Nmur2* transcript. RNAscope 4-plex negative (321831) and positive-control probes (321811) were processed in parallel with the target probes. All incubation steps were performed at 40 °C using the ACD HybEz hybridization system (321462) if not stated otherwise. One day before the assay, sections were mounted on SuperFrost Plus Gold slides (FT4981GLPLUS; Thermo Fisher), dried at room temperature (RT), briefly rinsed in autoclaved Millipore water, air dried and incubated at 60 °C for 4–6 h. Subsequently, slides were submerged in Target Retrieval reagent (322000) at 99.5 °C for 10 min, rinsed once in autoclaved Millipore water and dehydrated in 100% ethanol for 3 min. Slides were air dried for 5 min, a hydrophobic barrier was created around the sections using an ImmEdge hydrophobic barrier pen (310018) and slides were stored at RT until assaying. The following day, slides were incubated with Protease Plus (322330) for 25 min. The subsequent steps, that is, hybridization of the probes, amplification and detection steps, were performed according to the manufacturer’s protocol for RNAscope Fluorescent Multiplex Detection Reagent kit v2 (323110), or for more than three probes, the protocol for RNAscope 4-Plex Ancillary Kit for Multiplex Fluorescent Kit v2 (323120) was used. The probes were detected using tyramide-diluted Opal690 (1:2,000 dilution), Opal650 (1:1,500 dilution), Opal620 (1:1,000 dilution), Opal570 (1:1,000 dilution), Opal520 (1:750 dilution) or Cy3 (1:750 dilution). Sections were counterstained with DAPI and coverslipped with ProLong Gold Antifade Mountant (Thermo Fisher, P36931) and stored in the dark at 4 °C until imaged.

### Imaging and quantification of RNA ISH

Images were captured using a confocal Leica TCS SP-8-X microscope, equipped with a ×40/1.30 oil objective. Next, *z*-stacks were taken with optical sections of 0.9 μm. Laser intensities were kept constant throughout all related conditions. Images were imported into FIJI (National Institutes of Health, version 2.0.0-rc-41/1.50d), where maximum intensities were projected. For representative images, adjustments in brightness and contrast for each channel were kept constant throughout all related conditions, whereas for quantifications, all channels were kept unmodified and approximately five to ten sections were quantified per mouse and, if required, per area. For intensity quantification of endogenous *Lepr*, *Glp1r* and *Pomc* expression in C57BL/6N mice, all channels were imported and fused into the Halo software (Indica Labs, V.2.2.1870). The software relies on the DAPI stain for cellular identification and calculates the cell intensity for each cell and probe (a number integrating both the fluorescence intensity and the covered probe area within the designated cell). The threshold for probe recognition was determined by visual judgment, considering approximately five or more signals per cell as positive. For generation of the distribution pattern of POMC neuronal subpopulations throughout a coronal cross section, comparable anatomical locations within the ARC were analyzed correspondingly by the Halo software and three mice per replicate were merged. For quantification of *Cartpt*, *Npy1r*, *Oprm1* and *Nmur2* in C57BL/6N mice, integrated density was assessed via FIJI in previously defined single-cell ROIs showing *Pomc* signal and five or more signals of the *Lepr* or *Glp1r* probe. Cell counting of ROSA26rSrZsGreen and ROSA26lSlrSrhM3DGq mice was performed manually, defining single-cell ROIs showing *Pomc* and *ZsGreen* signal, and considering five or more probe signals per ROI of *Agrp*, *Lepr*, *Glp1r*, *Fos*, *Vglut2* or *Vgat* as positive.

### Gene copy number assay

For determining the *Pomc* copy number, 20 ng of genomic DNA extracted via standard isopropanol precipitation from tail biopsies was used. Selected gene segments were amplified using TaqMan Universal PCR-Master Mix (Thermo Fisher Scientific, 4305719) according to the manufacturer’s instructions. Non-exon-spanning primers (P1: 5′-GCGACAGGGACCAAACGG-3′, P2: 5′-AGACACCCTTACCTGTCGC-3′) and probes (5′-FAM-TCAGTGGCCTCTCTTAGTCACTGC-TAMRA-3′) were designed for amplification of *Pomc* exon 1, while the gene expression assay for *Socs3* (Thermo Fisher Scientific, Mm00545913_s1, 4331182) was purchased. The expression of *Pomc* exon 1 was normalized to *Socs3* mRNA. Results were calculated by the ΔCt comparative method (as 2^−ΔΔCt^), in which fold changes were calculated by defining wild-type animals with two *Pomc* gene copy numbers.

### uDISCO whole-brain clearing and image acquisition

The protocol was adapted from the uDISCO clearing method^22^. POMC^Dre^ ROSA26rSrZsGreen^+/−^, POMC^Dre^ Lepr^Cre^ ROSA26lSlrSrZsGreen^+/−^ and POMC^Dre^ Glp1r^Cre^ ROSA26lSlrSrZsGreen^+/−^ male and female mice were perfused with 1× PBS (pH 7.4) for 10 min followed by 4% PFA/PBS for 10 min. The brains were post-fixed for 24 h in 4% PFA/PBS (pH 7.4) at 4 °C. The brains were dehydrated in a gradient manner via incubations in tert-Butanol diluted in distilled water: 30%, 50%, 70%, 80%, 90% and 96% (vol) for 10–16 h at 37 °C. The brains were subsequently incubated in dichloromethane for 90–120 min at RT to remove lipids. Next, BABB-D4 was used as the reagent-matching solution for 12 h at RT to complete the clearing process (BABB: benzyl alcohol + benzyl benzoate at a 1:2 ratio, respectively; BABB-D4: a mixture of BABB and diphenyl ether (DPE) at a ratio 4:1 (vol/vol)). The cleared brains were imaged with a LaVision Bio Tec Ultramicroscope II with the acquisition software Inspector Pro (V.5.0.222.0). Whole-brain and magnified scans of the ARC were obtained using ×1.6 and ×8 total magnification, respectively. For the projection density analysis, cleared brains from POMC^Dre^ Lepr^Cre^ ROSA26lSlrSrtdTomato^+/−^ and POMC^Dre^ Glp1r^Cre^ ROSA26lSlrSrtdTomato^+/−^ mice were imaged using ×1.6 magnification with the same laser intensity across all samples.

### Whole-brain immunostaining

Pretreatment before staining was as follows: the fixed brains were washed two times in PBS for 1 h, incubated in 50% methanol (in PBS) for 1 h, 80% methanol for 1 h and two times in 100% methanol for 1 h. The samples were bleached with 5% H_2_O_2_ in 20% DMSO/methanol (one vol 30% H_2_O_2_/one vol DMSO/four vol methanol) at 4 °C overnight. Subsequently, samples were transferred to methanol for 1 h twice, then in 20% DMSO/methanol for 1 h twice, then in 80% methanol for 1 h, 50% methanol for 1 h, twice in PBS for 1 h, and finally in PBS/0.2% Triton X-100 for 1 h twice before further staining procedures. Pretreated samples were incubated in PBS/0.2% Triton X-100/20% DMSO/0.3 M glycine at 37 °C overnight, then blocked in PBS/0.2% Triton X-100/10% DMSO/6% donkey serum at 37 °C overnight. This was followed by a wash in PBS/0.2% Tween-20 with 10 μg ml^−1^ heparin (PTwH) for 1 h twice, then incubated in rat anti-mCherry (for tdTomato; Thermo Fisher Scientific, M11217; 1:500 dilution) in PTwH/5% DMSO/3% donkey serum at 37 °C for 8 d. After a 1-d long wash in PTwH, the samples were incubated in anti-rat Alexa Fluor 594 (Thermo Fisher Scientific, A21207; 1:300 dilution) in PTwH/3% donkey serum at 37 °C for 4 d. Samples were finally washed in PTwH before starting the clearing protocol, followed by LSFM imaging.

### Co-registration using VINCI

An automated workflow for co-registering each ×4-magnified scan per brain was registered to its full-brain image. Each full-brain image was subsequently registered to the Allen Brain 25-μm reference mouse brain atlas, using rigid-body correlation-coefficient and 12-parameter affine mutual information schemas in VINCI^23^. Originally, VINCI was developed for co-registration of clinical data but can also be utilized in animal studies^46^. The parameters were adapted for the multi-scale approach and preprocessing (quantile filtering) for microscopy data (VINCI versions 4.61.0 and 4.96.0). However, the size of the microscopy scans meant that further optimization was required; we needed to co-register 32-GB image files (8 × 10^9^ voxels), while typical-use cases in positron-emission tomography, magnetic resonance imaging and computed tomography for animal and human data are in the order of 2 × 10^7^ voxels. The processing of these large datasets was expedited through multi-threading, and the co-registration time on a dedicated system (56 cores) could be reduced from over 24 h to 0.7 h. The transformation matrix of the ×4 image to the atlas was derived as the combination of the two transformations. Quality of registration was determined based on a visual inspection of the registration result (a match of the interhemispheric fissures and the anatomical surface at the base of the hypothalamus). The scans with the best co-registration in each group were identified and the ×4 images from each mouse were aligned to the ×4 scan of the reference mouse within each group. This was performed using another specially adapted 12-parameter affine mutual information schema combined with the known transformation of the reference mouse full-brain image to the atlas. The correlation-coefficient values for the reference brain for POMC, Lepr and Glp1r were 0.85, 0.75 and 0.90, respectively. The quality of registration for the Glp1r group was not optimal so the Glp1r scans were co-registered to the ×4–×4 script but instead to the reference mouse of the POMC group.

### Extraction of neuronal coordinates using Arivis and three-dimensional scatterplots

The output NIFTI files of the co-registered ×4 brain scans were uploaded into the Arivis Vision 4D software (V.3.3.0). Individual neurons were identified using the Blob Finder feature in the software and their *xyz* coordinates were extracted. A Python script was used to visualize the 3D scatterplots for each neuronal group, where the quality of registration was checked once again. For the coronal view of the 3D distribution, the 3D scatterplots of three mice (per group) were plotted and a coronal snapshot was created from rostral locations within the ARC comparable to that of the two-dimensional (2D) coronal distribution. The coordinates of the 2D coronal distributions were also extracted using the Arivis software from the Halo output files (‘Imaging and quantification of RNA ISH’) that had been previously aligned manually. The final distribution graph was plotted using the Prism software.

### Isosurface density plots

To create the isosurface density plots, a Python script was used (V2.7.12). The probability density function of the 3D distribution of neurons was estimated for the whole POMC population, as well as the Lepr and Glp1r subpopulations. This was performed using the non-parametric kernel density estimation method. The density of neurons within the ARC was thus calculated based on the 3D neuronal coordinates to demonstrate the spatial concentration, which was then color coded to visualize the regions within the ARC with higher neuronal counts for each subpopulation.

### Data analysis of three-dimensional projections

Brain scans were co-registered to an annotated atlas (https://scalablebrainatlas.incf.org/mouse/ABA12#downloads/) using VINCI. Thresholding was achieved using the threshold function on VINCI by applying a value across all scans. Using the automated calculation function of VINCI, a mask was created from the ROIs in 3D, and the intensity profile of each region was determined using the VOI define function on VINCI. Representative images in the areas of interest were rendered using the Arivis software.

### Purification of mRNA from triple-positive EGFPL10a mice

The TRAP technique was performed using a modified version of a previous study on hypothalami of mice described under ‘Study design of bacTRAP (EGFPL10a) mice’^47^. One day before TRAP, 375 µl of Dynal Protein G magnetic beads (Invitrogen) was washed three times (1 ml) and resuspended in 275 µl of IP wash buffer (20 mM HEPES (pH 7.4), 150 mM KCl, 5 mM MgCl_2_ and 1% NP-40). Then, 50 µg of two monoclonal anti-GFP antibodies (HtzGFP-19C8 and HtzGFP-19F7) from the Memorial Sloan-Kettering Cancer Center was added to the magnetic beads and incubated with slow end-over-end mixing overnight at 4 °C.

On the day of TRAP, Dynal Protein G magnetic beads were washed three times (1 ml) and resuspended in 200 µl IP buffer (20 mM HEPES (pH 7.4), 150 mM KCl, 5 mM MgCl_2_, 1% NP-40, 0.5 mM dithiothreitol and 100 mg ml^−1^ cycloheximide) to remove unbound anti-GFP. Ice-cold polysome extraction buffer (20 mM HEPES (pH 7.4), 150 mM KCl, 5 mM MgCl_2_, 0.5 mM dithiothreitol, 100 mg ml^−1^ cycloheximide, protease inhibitors and 40 U ml^−1^ recombinant RNasin Ribonuclease inhibitor) was added to the samples containing mouse hypothalami and homogenized with a motor-driven Teflon glass homogenizer. Homogenates were centrifuged at 4 °C for 10 min at, 2000*g* to pellet cell debris. Supernatant was transferred to a new microcentrifuge tube and NP-40 (Applichem) and 1,2-diheptanoyl-*sn*-glycero-3-phosphocholine (DHPC; Avanti Polar Lipids) were added to the supernatant at a final concentration of 1% and 30 mM, respectively. After incubation on ice for 5 min, the clarified lysate was centrifuged for 10 min at 13,000*g* to pellet insoluble material. Next, 30 µl of supernatant (input) was snap frozen in liquid nitrogen until RNA extraction as a comparison to the immunoprecipitated sample. Then, 200 µl of anti-GFP-coated Dynal protein G magnetic beads was added to the supernatant, and the mixture was incubated at 4 °C with end-over-end rotation for 1 h. Beads were subsequently collected on a MagnaRack (Invitrogen), washed four times with high-salt polysome wash buffer (20 mM HEPES (pH 7.4), 350 mM KCl, 5 mM MgCl_2_, 1% NP-40, 0.5 mM dithiothreitol and 100 mg ml^−1^ cycloheximide). Input and IP beads were resuspended and incubated in RLT buffer (RNAeasy micro kit, Qiagen) for 5 mins at RT. Supernatant was removed from the IP beads and RNA extraction was performed according to Qiagen’s protocol, including in-column DNase digestion. RNA was resuspended in 10 μl nuclease-free water. RNA quantity and quality of input and IP were determined with a Qubit Fluorometer (Invitrogen) and Agilent 2100 Bioanalyzer.

### RNA sequencing

Pre-amplification was carried out using the Ovation RNA-seq system (V2). Total RNA was used for first-strand cDNA synthesis, using both poly(T) and random primers, followed by second-strand synthesis and isothermal strand-displacement amplification. For library preparation, the Illumina Nextera XT DNA sample preparation protocol was used, with 1 ng cDNA input. After validation (Agilent 2200 TapeStation) and quantification (Invitrogen Qubit System), transcriptome libraries were pooled. The pool was quantified using the Peqlab KAPA Library Quantification Kit and the Applied Biosystems 7900HT Sequence Detection and sequenced on an Illumina HiSeq 4000 sequencing instrument with a 2 × 75-bp paired-end read length protocol.

### Gene Ontology analysis

The regulated GO^48^ terms were derived using the clusterProfiler R package^49^ and visualized by mapping the percentage of regulated genes to the total GO term gene count against significant gene counts and adjusted *P* values per term. We visualized the GO term as a heat map, where each row represents a differentially expressed gene belonging to the term and each column represents a sample. To ensure visual differentiation between the rows not being skewed by highly expressed genes, the values of each sample were converted to *z*-scores with respect to the individual genes.

### Overlap analysis

We used publicly available single-cell RNA-seq data from mouse hypothalami to detect overlaps with markers identified in our Glp1r and Lepr datasets. The first data source used was single-cell RNA-seq of 20,921 cells from the arcuate–median eminence complex of mice^11^. We filtered cells expressing POMC (4,248/20,921 cells) and clustered them using the R Seurat package^24^. The clustering result was visualized using the UMAP plot of Seurat. The second data source used was from the single-cell RNA-seq dataset of 163 POMC-expressing neurons in mice yielding four clusters^12^.

### Electrophysiological experiments

#### Animals and brain slice preparation

Experiments were performed on brain slices from 12- to 15-week-old genetically marked (with ZsGreen) Glp1r- and Lepr-expressing POMC neurons using POMC^Dre^ Lepr^Cre^ ROSA26lSlrSrZsGreen^+/−^ or POMC^Dre^ Glp1r^Cre^ ROSA26lSlrSrZsGreen^+/−^ male and female mice. Animals were kept under standard laboratory conditions, with tap water and chow available ad libitum, on a 12 h light/dark cycle. The animals were lightly anesthetized with isoflurane (B506; AbbVie) and decapitated. Coronal slices (270–300 µm) containing the ARC were cut with a vibration microtome (HM-650 V; Thermo Scientific) under cold (4 °C), carbogenated (95% O_2_ and 5% CO_2_), glycerol-based modified artificial cerebrospinal fluid (GaCSF)^50^. GaCSF contained (in mM): 244 glycerol, 2.5 KCl, 2 MgCl_2_, 2 CaCl_2_, 1.2 NaH_2_PO_4_, 10 HEPES, 21 NaHCO_3_ and 5 glucose, adjusted to pH 7.2 with NaOH. If not mentioned otherwise, the brain slices were continuously superfused with carbogenated aCSF at a flow rate of ~2.5 ml min^−1^. aCSF contained (in mM): 125 NaCl, 2.5 KCl, 2 MgCl_2_, 2 CaCl_2_, 1.2 NaH_2_PO_4_, 21 NaHCO_3_, 10 HEPES and 5 glucose, adjusted to pH 7.2 with NaOH. To block GABAergic and glutamatergic synaptic input, in all recordings, the aCSF contained 10^−4^ M picrotoxin (P1675; Sigma-Aldrich), 5 × 10^−6^ M CGP (CGP-54626 hydrochloride; BN0597, Biotrend), 5 × 10^−5^ M DL-AP5 (DL-2-amino-5-phosphonopentanoic acid; BN0086, Biotrend) and 10^−5^ M CNQX (6-cyano-7-nitroquinoxaline-2,3-dione; C127, Sigma-Aldrich). To suppress action-potential-dependent synaptic release, blocked voltage-dependent Na^+^ channels were blocked by 10^−6^ M TTX (T-550, Alomone).

#### Electrophysiology

Current-clamp and voltage-clamp recordings of ZsGreen-expressing POMC neurons were performed at ~32 °C in the perforated patch-clamp configuration. Neurons were visualized with a fixed-stage upright microscope (BX51WI, Olympus) using ×40 and ×60 water-immersion objectives (LUMplan FL/N ×40, 0.8 numerical aperture, 2 mm working distance; LUMplan FL/N ×60, 1.0 numerical aperture, 2 mm working distance, Olympus) with infrared differential interference contrast optics^51^ and fluorescence optics. ZsGreen-expressing POMC neurons were identified by their anatomical location in the ARC and by their ZsGreen fluorescence that was visualized with an X-Cite 120 illumination system (EXFO Photonic Solutions) in combination with a Chroma 41001 filter set (ex: HQ480/×40; bs: Q505LP; em: HQ535/50m). Electrodes with tip resistances of between 4 and 6 MΩ were fashioned from borosilicate glass (0.86-mm inner diameter; 1.5-mm outer diameter; GB150-8P, Science Products) with a vertical pipette puller (PP-830, Narishige). All recordings were performed with an EPC10 patch-clamp amplifier (HEKA) controlled by the program PatchMaster (version 2.32; HEKA) running in Windows. In parallel, data were recorded using a micro1410 data acquisition interface and Spike 2 (version 7, both from CED). Current-clamp recordings were sampled at 25 kHz and low-pass filtered at 2 kHz with a four-pole Bessel filter. Voltage-clamp recordings were sampled at 5 kHz, smoothed (𝜏 = 0.2 s) and downsampled to 0.5 Hz. The calculated liquid junction potential of 14.6 mV between intracellular and extracellular solution was compensated or subtracted offline (calculated with Patcher’s Power Tools plug-in from https://www3.mpibpc.mpg.de/groups/neher/index.php?page=software for IGOR Pro 6; Wavemetrics).

Perforated patch experiments were conducted using protocols modified from previous studies^52,53^. Recordings were performed with pipette solution containing (in mM): 140 K-gluconate, 10 KCl, 10 HEPES, 0.1 EGTA and 2 MgCl_2_, adjusted to pH 7.2 with KOH. ATP and GTP were omitted from the intracellular solution to prevent uncontrolled permeabilization of the cell membrane^54^. The patch pipette tip was filled with internal solution and backfilled with internal solution, which contained the ionophore to achieve perforated patch recordings and 0.02% tetramethylrhodamine-dextran (3,000 MW, D3308, Invitrogen) to monitor the stability of the perforated membrane. Amphotericin B (A4888; Sigma) was dissolved in DMSO to a concentration of 40 µg µl^−1^ (D8418, Sigma) following the protocols of a previous study^55^. The used DMSO concentration (0.1–0.3%) had no obvious effect on the investigated neurons. The ionophore was added to the modified pipette solution shortly before use. The final concentration of amphotericin B was ~120–160 µg ml^−1^. Amphotericin solutions were prepared from undissolved weighted samples (stored at 4 °C protected from light) on every recording day. During the perforation process, access resistance (*R*_a_) was monitored continuously and experiments started after *R*_a_ values reached steady state (~15–20 min) and the action potential amplitude was stable.

#### Intrinsic electrophysiological properties

To analyze in detail the intrinsic electrophysiological properties of ZsGreen-expressing POMC neurons, a set of current-clamp protocols from a holding potential of −70 mV was applied. Cell input resistance was determined from a series of hyperpolarizing small current pulses (1 s, 2–10 pA increments) and the slope of the resulting I–V relations. Whole-cell capacitances were calculated from the membrane time constant (𝜏) and the input resistance (R): C = 𝜏/R. To analyze the *I*_H_-dependent sag potentials, the neurons were hyperpolarized with five consecutively incrementing current pulses. The increments were adjusted so that the last pulse hyperpolarized the membrane to −120 mV. The sag potential was defined as the difference between the lowest voltage reached at the beginning of the pulse and the membrane potential reached at the end of hyperpolarization. To analyze post-inhibitory rebound excitation, we used an ‘enhanced rebound protocol’, whereby the same current-step amplitudes were applied as those used for the sag-potential analysis, but this time as 2-s hyperpolarizing pre-pulses that were followed by a 1-s test pulse with the amplitude of a single increment. The maximum instantaneous frequencies during the rebound were determined and plotted over the membrane potentials of the pre-pulses. To analyze input–output relations, we applied a series of ascending and then descending current ramps (5 s each), where the ramp amplitudes were increased from 10 to 25 pA in 5-pA increments. Amplitudes were further increased if the 25-pA ramp did not elicit action potentials. Spike-number ratios were calculated by dividing the number of action potentials during the ascending ramp by the number of action potentials during the descending ramp. To further analyze excitability, that is, evoked action potential firing, a series of depolarizing current pulses (1 s; 5–50 pA in 5-pA increments) was applied. For each current pulse, the number of action potentials was determined, plotted over the current amplitude and linearly fit. Linear fits were performed for data points where action potentials were elicited. Only data points at which action potentials were triggered were considered for fit.

For SFA ratios, 10-s depolarizing stimuli were applied from a holding potential of −70 mV with initial instantaneous action potential frequencies between 30 and 40 Hz. Instantaneous frequencies were plotted (*Y*) over the 10-s time course, and fit to a mono-exponential decay equation with Y_0_ set to the initial instantaneous frequency: *Y* = (*Y*_0_ − plateau) × exp(−K × *T*) + plateau, where ‘plateau’ is the asymptotic frequency, K is the inverse time constant and *T* is the time. The SFA ratio is determined by dividing the maximum initial instantaneous frequency by the plateau frequency of the fit. Action potential waveform parameters were obtained from action potentials with instantaneous frequencies ≤ 5 Hz. If necessary, hyperpolarizing bias currents were used to decrease spontaneous firing.

#### Peptide signaling

Leptin (100 nM; L3772, Sigma-Aldrich), and Glp1 (300 nM; H-5956, Bachem AG) were bath applied for 15 or 30 min with a perfusion rate of 2.5 ml s^−1^. Npy (100 nM; N5071, Sigma-Aldrich) was bath applied for 10 min.

#### Leptin and Glp1 signaling

In line with previous studies, we found that the basic firing properties of POMC neurons and their responsiveness to leptin and Glp1 were not homogeneous. Therefore, we used the ‘three times standard deviation’ (3σ) criterion, and a neuron was considered responsive if the change in firing frequency or membrane potential induced by leptin or Glp1 was three times larger than the standard deviation. Means and respective standard deviations of spontaneous action potential firing or membrane potential were calculated from a period of 120 s, divided into 12 bins, each 10 s long. Data were taken immediately before and at the end of the peptide application.

#### Voltage-clamp recording of peptide-induced currents

The NPY action on POMC neurons was analyzed under voltage clamp. Based on previous studies^56–58^ and on the peptide-induced action-potential frequency modulation, which we observed in current-clamp recordings, the holding potential was set to −55 mV to optimize the recording conditions to measure inward currents. NPY (100 nM) was bath applied for 10 min after a 5-min baseline recording. The recorded peptide-induced currents were baseline subtracted and the mean (±s.e.m.) was calculated. To quantify differences in the peptide-induced currents, the area under the curve (electrical charge in nC) during the 10-min application of NPY was calculated.

### Quantification and statistical analysis

#### Statistical analyses and reproducibility

Details on statistical analysis for Figs. 3, 5, 7 and 8 can be found in ‘Statistical analysis of 3D data’, ‘RNA-sequencing analysis workflow’ and ‘Electrophysiological data analysis and statistics’, respectively. Primary data processing and organization were performed in Microsoft Excel (2010). Statistical analyses were performed using Prism software (GraphPad, V.5.0–V.8.0). Statistical significance for two groups was determined by unpaired two-tailed Student’s *t*-test. In the case of unequal variance between the two groups, the unpaired Welch’s *t*-test or unpaired Mann–Whitney *U*-test was used. For determining differences between more than two groups, one-way ANOVA was applied. Depending on the scientific question, one-way ANOVA was followed by no post hoc test, by Dunnett’s post hoc test (comparing all groups to one control) or by Tukey’s post hoc test (comparing all groups among each other), as indicated in the figure legends. All remaining data (more than two groups, more than one independent factor) were analyzed with two-way ANOVA followed by Sidak’s or Tukey’s post hoc analysis. Data are expressed as the mean, and the error bars indicate the s.e.m. unless specified otherwise. In the violin plots, solid lines represent median values, and dashed lines represent lower and upper quartiles. A detailed description of statistics, including individual data points, tests, *P* values and further statistical parameters, are provided in the figure legends and as source data. Statistical significance was defined as: *P ≤ 0.05, **P ≤ 0.01, ***P ≤ 0.001 and ****P ≤ 0.0001.

No statistical methods were used to predetermine sample sizes, although sample sizes are similar to those reported in previous publications^6,39,59^. Data collection and analysis were carried out in a blinded format throughout the study, unless this was not possible due to the visual differences in cases of varying neuronal numbers resulting from genetic labeling as depicted in Fig. 3a–c. Data distribution was assumed to be normal, but this was not formally tested. Except for animals that died during an experiment, no data were excluded. For metabolic phenotyping, every mouse represents a replicate (*n*) and the number of replicates is mentioned for each experiment in the figure legend and/or source data. In this case, data were pooled from independent experiments of varying *n* numbers. For RNA-seq, samples of pooled hypothalami were collected from individual mice of several cohorts. For electrophysiological experiments, the sample numbers indicate the number of cells used in recordings. All measurements that did not require statistical analysis, such as representative images, were obtained from at least two animals and in most cases a minimum of three animals were used.

#### Statistical analysis of three-dimensional data

To carry out a statistical assessment of the differences in distribution patterns for the two subpopulations, the bounding box containing all the neuronal coordinates was divided into smaller cubes and two-sided Student’s *t*-test was calculated for each cube using a Python script (V.2.7.12). Due to the variance in the total neuronal counts between the Lepr and Glp1r groups, the number of neurons in each cube was corrected by introducing a density factor (number of Glp1r neurons/number of Lepr neurons). This was performed by multiplying the density factor to the total number of neurons in each group. Since the null distribution is two-sided, both null hypotheses were visualized within the same plot.

### RNA-sequencing analysis workflow

The RNA-seq results of Glp1r and Lepr samples were processed using the nf-core/rnaseq pipeline (V.1.4.2)^60^. This involves (1) aligning the raw reads to the reference genome GRCm38.p6 using STAR (2.6.1d)^61^ and (2) transcript abundance estimation using Salmon (0.14.1)^62^ with the reference transcriptome from Ensembl (release 97)^63^. To normalize the ribosomal pulldown (IP) to the hypothalamic background (input) per sample, we calculated a ratio of the Salmon gene counts (IP/input). The differentially expressed genes between Glp1r and Lepr groups were derived using DESeq2 (V.1.26.0)^64^ where *P* values attained by the Wald test were corrected for multiple testing using the Benjamini–Hochberg method. This yielded genes that are regulated between the Glp1r and Lepr conditions and act as markers for these POMC populations.

### Electrophysiological data analysis and statistics

Data analysis was performed with Spike2 (V.7.0; Cambridge Electronic Design), Igor Pro 6 (Wavemetrics) and GraphPad Prism (V.5.0-8.0). In violin plots, the solid line represents the median, and dashed lines represent lower and upper quartiles. In the box plots, the ‘+’ sign depicts the mean, and the horizontal line represents the median. The whiskers were calculated according to Tukey’s method. For pairwise comparisons of dependent and independent normal distributions, paired and unpaired *t*-tests were used, respectively. For pairwise comparisons of independent, non-normal distributions, a Mann–Whitney *U*-test was used. For multiple comparisons, one-way ANOVA with post hoc Tukey’s test was performed. A significance level of 0.05 was accepted for all tests. In the figures, *n* values are given in parentheses. Exact *P* values are reported if *P* > 0.05.

### Reporting Summary

Further information on research design is available in the Nature Research Reporting Summary linked to this article.

## Online content

Any methods, additional references, Nature Research reporting summaries, source data, extended data, supplementary information, acknowledgements, peer review information; details of author contributions and competing interests; and statements of data and code availability are available at 10.1038/s41593-021-00854-0.

## Supplementary information

Reporting Summary

## Data Availability

Raw RNA-seq data have been deposited in the NCBI Gene Expression Omnibus under accession code GSE153753. Other raw data is available under reasonable request to the corresponding author.

## Source data

Source Data Extended Data Fig. 1 Statistical details. Source Data Extended Data Fig. 3 Statistical details. Source Data Extended Data Fig. 5 Statistical details. Source Data Extended Data Fig. 6 Statistical details. Source Data Extended Data Fig. 7 Statistical details. Source Data Extended Data Fig. 8 Statistical details. Source Data Extended Data Fig. 10 Statistical details.

## Code availability

The code for RNA-seq analysis is available at https://github.com/bruening-lab/pomc-neurons-architecture-rnaseq/ and the code for isosurface density plots and the 3D statistical analysis is available at https://github.com/bruening-lab/Heterogeneity_Scripts/. Source data are provided with this paper.
