# Supplementary material for: Functionally distinct POMC-expressing neuron subpopulations in hypothalamus revealed by intersectional targeting
Source: Nat Neurosci. 2021 May 17;24(7):913–29. doi: 10.1038/s41593-021-00854-0 (PMC8249241; doi:10.1038/s41593-021-00854-0)
Supplement: Supplementary file 1 — Reporting Summary [file 41593_2021_854_MOESM1_ESM.pdf]

nature research

Corresponding author(s): Jens C. Bruening

Last updated by author(s): 10.02.21

Reporting Summary

Nature Research wishes to improve the reproducibility of the work that we publish. This form provides structure for consistency and transparency in reporting. For further information on Nature Research policies, see our Editorial Policies and the Editorial Policy Checklist.

Statistics

For all statistical analyses, confirm that the following items are present in the figure legend, table legend, main text, or Methods section.

n/a	Confirmed	
☐	☒	The exact sample size (n) for each experimental group/condition, given as a discrete number and unit of measurement
☐	☒	A statement on whether measurements were taken from distinct samples or whether the same sample was measured repeatedly
☐	☒	The statistical test(s) used AND whether they are one- or two-sided<br>*Only common tests should be described solely by name; describe more complex techniques in the Methods section.*
☒	☐	A description of all covariates tested
☐	☒	A description of any assumptions or corrections, such as tests of normality and adjustment for multiple comparisons
☐	☒	A full description of the statistical parameters including central tendency (e.g. means) or other basic estimates (e.g. regression coefficient) AND variation (e.g. standard deviation) or associated estimates of uncertainty (e.g. confidence intervals)
☐	☒	For null hypothesis testing, the test statistic (e.g. F, t, r) with confidence intervals, effect sizes, degrees of freedom and P value noted<br>*Give P values as exact values whenever suitable.*
☒	☐	For Bayesian analysis, information on the choice of priors and Markov chain Monte Carlo settings
☒	☐	For hierarchical and complex designs, identification of the appropriate level for tests and full reporting of outcomes
☒	☐	Estimates of effect sizes (e.g. Cohen's d, Pearson's r), indicating how they were calculated

Our web collection on statistics for biologists contains articles on many of the points above.

Software and code

Policy information about availability of computer code

Data collection	Image acquisition software: Leica ASX v.3.5.5.19976, Imspector Pro v.5.0.222.0.<br>Indirect calorimetry data acquisition was carried out using TSE Phenomaster versions 6.2.5 and above.<br>For data acquisition on an IVIS Spectrum CT scanner (Caliper LifeScience, USA) we used IVIS LivingImage Software V4.3.1.
Data analysis	Details are provided in the Methods section. The main software used are as follows:<br>VINCI (versions 4.61.0 and 4.96.0) was used for co-registration of the mouse brain scans acquired using light sheet fluorescent microscopy.<br>Arivis Vision 4D (versions 2.12 and 3.3.0) was used for 3D rendering, visualization and neuronal coordinate extraction.<br>Custom-written Python (version 2.7.12) script was used to create isosurface density plots and to carry out statistical analysis of the 3D data. The RNA-sequencing results were processed using the nf-core/rnaseq pipeline v1.4.2. Both aforementioned codes are available on Github. Confocal images were mainly analyzed by FIJI (NIH, version 2.0.0-rc-41/1.50d). For intensity quantification of endogenous Lepr, Glp1r and Pomc expression in C57BL/6N mice, the Halo software (Indica Labs, V.2.2.1870) was used.<br>Data analysis was also performed using Spike2 (version 7; Cambridge Electronic Design Ltd., Cambridge, UK), Igor Pro 6 (Wavemetrics, Portland, OR, USA), and Graphpad Prism (versions 5.0-8.0; Graphpad Software Inc., La Jolla, CA, USA).

For manuscripts utilizing custom algorithms or software that are central to the research but not yet described in published literature, software must be made available to editors and reviewers. We strongly encourage code deposition in a community repository (e.g. GitHub). See the Nature Research guidelines for submitting code & software for further information.

Data

Policy information about availability of data

All manuscripts must include a data availability statement. This statement should provide the following information, where applicable:

- Accession codes, unique identifiers, or web links for publicly available datasets
- A list of figures that have associated raw data
- A description of any restrictions on data availability

Raw RNA-Seq data have been deposited into the NCBI Gene Expression Omnibus (GEO) under the accession code: GSE153753. Raw data is available under reasonable request to the corresponding author.
The code for RNA-sequencing analysis is available at https://github.com/bruening-lab/pomc-neurons-architecture-rnaseq and the code for isosurface density plots as well as the 3D statistical analysis is available at https://github.com/bruening-lab/Heterogeneity_Scripts
Ensembl release 97 was used for reference genome, transcripts and GO-term analysis. In the comparison of the results of our RNA sequencing with publicly available datasets we used data published by Campbell et al. (2017), as well as the study by Yeo et al. (2017).

Field-specific reporting

Please select the one below that is the best fit for your research. If you are not sure, read the appropriate sections before making your selection.

☒ Life sciences ☐ Behavioural & social sciences ☐ Ecological, evolutionary & environmental sciences

For a reference copy of the document with all sections, see nature.com/documents/nr-reporting-summary-flat.pdf

Life sciences study design

All studies must disclose on these points even when the disclosure is negative.

Sample size	No statistical methods were used to pre-determine sample sizes and the sample sizes in the study are similar to those reported previously: Brandt et al. (2018), Vogt et al. (2015), Konner et al. (2007).
Data exclusions	Except for animals that died during the course of an experiment, no data were excluded.
Replication	For metabolic phenotyping, every mouse represents a replicate (n) and the number of replicates is mentioned for each experiment in the figure legend and/or supporting materials. In this case, data was pooled from independent experiments of varying n numbers. For RNA sequencing, samples of pooled hypothalami were collected from individual mice of several cohorts. For electrophysiological experiments, the sample numbers indicate the number of cells recorded from. All measurements that didn't require statistical analysis such as representative images were obtained from at least 2 animals and in most cases a minimum of 3 animals were used.
Randomization	Mice used for experiments were assigned to their corresponding experimental group by genotype. In case of treatments within one genotype, mice were assigned randomly to the treatment groups.
Blinding	Data collection and analysis were carried out in a blinded format throughout the study, unless this was not possible due to the visual differences in cases of varying neuronal numbers resulting from genetic labeling as depicted in Figure 3A-C.

Reporting for specific materials, systems and methods

We require information from authors about some types of materials, experimental systems and methods used in many studies. Here, indicate whether each material, system or method listed is relevant to your study. If you are not sure if a list item applies to your research, read the appropriate section before selecting a response.

Materials & experimental systems

n/a	Involved in the study	
☐	☒	Antibodies
☒	☐	Eukaryotic cell lines
☒	☐	Palaeontology and archaeology
☐	☒	Animals and other organisms
☒	☐	Human research participants
☒	☐	Clinical data
☒	☐	Dual use research of concern

Methods

n/a	Involved in the study	
☒	☐	ChIP-seq
☒	☐	Flow cytometry
☒	☐	MRI-based neuroimaging

Antibodies

Antibodies used	Primary antibodies: rabbit anti-ZsGreen (Takara Bio Clontech #632474, 1:100), rabbit anti-POMC (1:1000, Phoenix, #H-029-30), rat anti-mCherry (1:1000

Thermo Fisher Scientific, #M11217) and chicken anti-GFP (1:1000 Abcam, #ab13970)

Secondary antibodies:
donkey anti-rabbit-Alexa488 (ThermoFisher #A21206, 1:500), donkey anti-rat-594 (JacksonImmunoResearch, #712-585-153, 1:500) or goat anti-rabbit-594 (Thermo Fisher Scientific, #A11012, 1:500) and goat anti-chicken-488 (Thermo Fisher Scientific, #A11039, 1:500)

Validation

rabbit anti-ZsGreen (Takara Bio Clontech #632474)
https://www.takarabio.com/products/antibodies-and-elisa/fluorescent-protein-antibodies/green-fluorescent-protein-antibodies?catalog=632474

rabbit anti-POMC (Phoenix, #H-029-30)
https://www.phoenixpeptide.com/products/view/Antibodies/H-029-30

rat anti-mCherry (Thermo Fisher Scientific, #M11217)
https://www.thermofisher.com/antibody/product/mCherry-Antibody-clone-16D7-Monoclonal/M11217

chicken anti-GFP (Abcam, #ab13970)
https://www.abcam.com/gfp-antibody-ab13970.html

donkey anti-rabbit-Alexa488 (ThermoFisher #A21206)
https://www.thermofisher.com/order/genome-database/details/antibody/A-21206.html

donkey anti-rat-594 (JacksonImmunoResearch, #712-585-153)
https://www.jacksonimmuno.com/catalog/products/712-585-153

goat anti-rabbit-594 (Thermo Fisher Scientific, #A11012)
https://www.thermofisher.com/antibody/product/Goat-anti-Rabbit-IgG-H-L-Cross-Adsorbed-Secondary-Antibody-Polyclonal/A-11012

goat anti-chicken-488 (Thermo Fisher Scientific, #A11039)
https://www.thermofisher.com/antibody/product/Goat-anti-Chicken-IgY-H-L-Secondary-Antibody-Polyclonal/A-11039

Animals and other organisms

Policy information about studies involving animals; ARRIVE guidelines recommended for reporting animal research

Laboratory animals

For all experiments, male and female mice, between the ages of 4 to 26 weeks from the C57BL/6N background were used. Mice were housed in individually ventilated cages (IVCs) at 22°C–24°C using a 12 hr light/dark cycle. Animals had access to water and food ad libitum. All animal experiments were performed in accordance with regulations of the relevant animal welfare acts and protocols approved by the respective regulatory authorities.
LeprCre mice (Leshan et al. 2006) and the Glp1rCre (Richards et al. 2014) lines were kindly provided by Prof. MG. Myers and Prof. F. Reimann, respectively. The ROSA26rSrlSltdTomato was purchased from Jackson Laboratories. The POMCDre recombinase line and ROSA26lSlrSrhM3Dq, ROSA26lSlrSrEGFPL10a mouse lines were newly generated in this study.

Wild animals

No wild animals were assessed in this study.

Field-collected samples

No field collected samples were assessed in this study.

Ethics oversight

All animal procedures were conducted in compliance with protocols approved by local government authorities (Bezirksregierung Köln). Permissions for experiments and to maintain and breed mice was issued by the Department for Environment and Consumer Protection-Veterinary Section, Cologne, North Rhine-Westphalia, Germany.

Note that full information on the approval of the study protocol must also be provided in the manuscript.
